# Botanical Hybrid BioEntities with Antibacterial Activity Derived from *Geranium robertianum*—Biophysical Insights

**DOI:** 10.3390/biomimetics11100713

**Published:** 2026-10-06

**Authors:** Marcela-Elisabeta Barbinta-Patrascu, Irina Negut, Cornelia Nichita, Bogdan Bita, Vlad-Andrei Antohe, Georgiana-Alexandra Grigore, Diana-Madalina Gaboreanu, Adrian Bobica

**Affiliations:** 1 Department of Electricity, Solid-State Physics and Biophysics, Faculty of Physics, University of Bucharest, 405 Atomistilor, 077125 Magurele, Romania; marcela.barbinta@unibuc.ro (M.-E.B.-P.); bogdan.bita@inoe.ro (B.B.); vlad.antohe@fizica.unibuc.ro (V.-A.A.); 2 National Institute for Laser, Plasma and Radiation Physics, 409 Atomistilor, 077125 Magurele, Romania; 3 CTT-3Nano-SAE Research Center, Faculty of Physics, University of Bucharest, ICUB, 405 Atomistilor Street, P.O. Box MG-38, 077125 Magurele, Romania; cornelia.nichita@unibuc.ro; 4 National Institute for Chemical—Pharmaceutical Research and Development, 112 Vitan Avenue, 031299 Bucharest, Romania; 5 Optospintronics Department, National Institute of Research and Development for Optoelectronics-INOE 2000, 409 Atomistilor, 077125 Magurele, Romania; 6 Faculty of Biology, University of Bucharest, Splaiul Independentei 91–95, 050095 Bucharest, Romania; grigore.georgiana-alexandra@s.bio.unibuc.ro (G.-A.G.); gaboreanu.diana-madalina@s.bio.unibuc.ro (D.-M.G.); 7 Research Institute of the University of Bucharest—ICUB, University of Bucharest, 050663 Bucharest, Romania; 8 National Institute of Research and Development for Biological Sciences, Splaiul Independentei 296, 060031 Bucharest, Romania; 9 CROMATEC PLUS SRL, 1 Petre Ispirescu Street, Tâncăbeşti, 077167 Ilfov, Romania; adrian.bobica@cromatecplus.ro

**Keywords:** phyto-nano-silver, botanical synthesis, biomimetic membranes, *green* strategy, biohybrids, *Geranium robertianum* L., antioxidant activity, antibacterial properties

## Abstract

The present study reports the development of a novel botanical hybrid system obtained through the integration of *Geranium robertianum* L. phytochemicals, green-synthesized silver nanoparticles, and chlorophyll-loaded soybean lecithin biomimetic membranes. The proposed strategy combines the reducing and stabilizing capacity of plant-derived metabolites with the structural and functional advantages of membrane-inspired lipid assemblies. Silver nanoparticles were synthesized using aqueous *Geranium robertianum* extract, while biomimetic lipid vesicles were prepared from soybean lecithin and subsequently assembled into hybrid bioentities through a self-organization process. The obtained structures were characterized by UV–Vis spectroscopy, fluorescence spectroscopy, FTIR–ATR analysis, dynamic light scattering, zeta potential measurements, scanning electron microscopy, atomic force microscopy, and energy-dispersive X-ray spectroscopy. Optical investigations support the formation of phyto-capped silver nanoparticles and their successful incorporation into chlorophyll-containing biomimetic membranes. The hybrid structures exhibited improved colloidal stability, appropriate nanoscale dimensions, and characteristic morphological features indicative of efficient integration of the lipid and metallic components. Antioxidant and antibacterial investigations highlighted the combined contribution of the phytochemicals, silver nanostructures, and phospholipid matrix. These findings establish a versatile, environmentally friendly platform that bridges phytochemistry, nanotechnology, and biomimetic engineering, and may provide new opportunities for the development of multifunctional antimicrobial systems.

## 1. Introduction

*Geranium robertianum* L. (*G. robertianum*), commonly known as Robert Herb or Red Robin, and traditionally referred to as “Năpraznic” in Romania, is an annual or biennial medicinal plant widely distributed throughout Europe, Asia, and North Africa. The species has long been employed in traditional medicine for the treatment of inflammatory disorders, gastrointestinal diseases, diabetes, infections, and cancer-related conditions [1]. In recent years, increasing attention has been paid to *G. robertianum* owing to its rich phytochemical composition and broad spectrum of biological activities, which support many of its ethnopharmacological applications [1].

Phytochemical investigations have demonstrated that *G. robertianum* is particularly rich in polyphenolic constituents, including hydrolysable tannins, flavonoids, and phenolic acids [1,2]. Among these compounds, geraniin represents one of the characteristic ellagitannins of the species and is considered a major contributor to its biological activity. Other frequently reported constituents include ellagic acid, gallic acid, chlorogenic acid, caffeic acid, rutin, quercetin, kaempferol derivatives, and numerous flavonol glycosides [1,3]. These metabolites are recognized for their strong antioxidant properties and their involvement in multiple cellular protective mechanisms. One of the most extensively investigated bioactivities of *G. robertianum* is its antioxidant potential. Extracts from aerial parts exhibit pronounced free radical scavenging capacity, reducing power and protection against oxidative damage, largely attributed to their high phenolic content [1,3]. Furthermore, several studies have reported anti-inflammatory effects associated with the modulation of oxidative stress pathways and the scavenging of reactive oxygen and chlorine species involved in inflammatory processes [2]. The plant also displays significant antimicrobial properties. Recent studies demonstrated that optimized ethanolic and acetonic extracts exert broad-spectrum antibacterial activity against both Gram-positive and Gram-negative bacteria, with evidence of synergistic interactions with conventional antibiotics such as gentamicin. These findings suggest a potential role of *G. robertianum* extracts as adjuvant agents in the management of antimicrobial resistance [4]. In addition to its antimicrobial effects, *G. robertianum* has attracted considerable interest due to its antiproliferative and anticancer potential. Phenolic-rich extracts and fractions have shown cytotoxic activity against several human tumor cell lines, including hepatocellular, breast, cervical, and lung carcinoma models [3]. These effects have been associated with the induction of apoptosis, inhibition of cellular proliferation and reduction in oxidative stress. Recent investigations further support the contribution of polyphenols and flavonoids to the antitumor activity of the species, highlighting its potential as a source of novel bioactive compounds for cancer research [3]. Additional pharmacological studies have revealed promising antidiabetic and neuroprotective properties. Oral administration of *G. robertianum* decoctions in diabetic Goto–Kakizaki rats resulted in reduced blood glucose levels and improved mitochondrial oxidative phosphorylation efficiency, supporting its traditional use in diabetes management [5]. Moreover, aqueous extracts demonstrated neuroprotective effects in cellular models of Parkinson’s disease by attenuating oxidative damage, reducing apoptosis, and improving neuronal cell survival [6]. More recently, a comparative investigation of wild-grown Romanian *Geranium* species showed that *G. robertianum* had particularly high total phenolic content and antioxidant capacity, with DPPH, and ABTS assays supporting the contribution of its phenolic profile to antioxidant activity [7].

Overall, current evidence identifies *G. robertianum* as a valuable source of biologically active polyphenols with antioxidant, anti-inflammatory, antimicrobial, antidiabetic, neuroprotective, and anticancer properties. These findings provide a strong scientific rationale for further investigation of this species and support its potential development as a source of phytopharmaceutical and nutraceutical products [1]. To date, *Geranium robertianum* L. has been used for green synthesis of ZnO NPs [8]. To our knowledge, no study regarding the ecosynthesis of AgNPs using aqueous extract of *G. robertianum* has been reported. The *G. robertianum* plant was selected for our study due to its antioxidant and antimicrobial properties, as mentioned above.

Although green-synthesized silver nanoparticles (AgNPs) and lipid-based nanocarriers have been extensively investigated separately, integrating phyto-derived AgNPs, chlorophyll-loaded biomimetic membranes, and *G. robertianum* phytochemicals into biohybrid architectures remains largely unexplored. The present work addresses this challenge by developing a botanical-derived hybrid platform in which phytochemicals, metallic nanostructures, and phospholipid vesicles mimicking the fundamental lipid-bilayer organization of biological membranes act as complementary functional building blocks. To the best of our knowledge, this is the first study reporting chlorophyll-loaded soybean lecithin biomimetic membranes integrated with *G. robertianum*-derived AgNPs and systematically characterized through a combination of optical, colloidal, morphological, topographical, compositional and biological analyses. The resulting hybrid architecture exemplifies a biomimetic design strategy that bridges plant nanotechnology and membrane-inspired materials, providing a multifunctional platform with promising antioxidant and antibacterial potential.

The rationale for bio-design of the hybrid system is based on the bioactivities of their components. In this research, the aqueous extract of aerial parts of *G. robertianum* was used both as a reducing agent for silver ions and as capping agents for developed AgNPs. The phyto-generated particles were functionalized with biomimetic membranes labeled with chlorophyll—a natural optical sensor with antioxidant and antimicrobial properties [9,10].

As is widely known, Ag NPs synthesized using vegetal extracts (phyto-AgNPs) possess interesting bioactivities (such as antioxidant and antimicrobial), which are primarily due to the presence, on the surface of the AgNPs, of biomolecules derived from the precursor phyto-extract that exhibit antioxidant and antimicrobial activities [11]. Another key role in conferring bioactivities to nanomaterials is played by the nanosize effect, taking into account the fact that they are closely related to their high surface to volume ratio [12,13]. Moreover, capping agents play a vital role in preventing aggregation of phyto-AgNPs, by attaching to the surface of AgNPs [14], thus enhancing their stability.

Many studies have recently been conducted on the bioactivities of phyto-AgNPs. Thus, Lite et al. used aqueous extract of *Crataegus monogyna* [15] to synthesize AgNPs with strong performance against *Staphylococcus aureus* and antioxidant effects against both short-lived and long-lived free radicals. Marukurti et al. biosynthesized AgNPs by using methanolic leaf extract of *Plumeria rubra* [16] exhibited antioxidant properties and antimicrobial activity was tested against 6 pathogenic organisms. Another research group reported the use of aqueous leaf extract of *Perilla frutescens* (L.) obtained AgNPs with remarkable antioxidant activity, high antibacterial effects against *E. coli* and *S. aureus*, good antifungal activity against *C. albicans*, and also moderate toxicity on MCF-7 breast cancer cells [17].

Moreover, the biocomposites/biohybrids containing phyto-AgNPs possess novel properties and functionalities that open up new potential biomedical applications [18]. This is the reason why we developed biohybrids with biomimetic membranes (soybean lecithin membranes) in this study. The artificial cell membranes will help protect and enhance the dispersion and delivery of the incorporated AgNPs-GR. The liposomal system is biocompatible and biodegradable, and it enhances the therapeutic effect by facilitating interaction with natural biological membranes [19].

In this research study, the resulting biohybrid AgNPs/biomimetic membranes were investigated by various biophysical methods. The antioxidant and antibacterial properties have been assessed in order to investigate the potential bio-applications.

## 2. Materials and Methods

### 2.1. Materials

Folin–Ciocâlteu’s phenol reagent, 3,4,5-trihydroxybenzoic acid (gallic acid, >97.5% purity), sodium carbonate (≥99.0% purity), 2,2-Diphenyl-1-picrylhydrazyl (DPPH, 85% purity), hydrogen peroxide (30% (*w*/*w*)), 5-Amino-2,3-dihydro-1,4-phthalazinedione (luminol, ≥97.0% purity), TRIS-HCl buffer, and methanol (≥99.8%) were provided by Sigma-Aldrich (St. Louis, MO, USA). Ethanol (EtOH, 46.07 Da, ≥99.8%) and chloroform (CHCl_3_, 119.38 g/mol; ≥99.9%) were purchased from Merck Company (Darmstadt, Germany). Soybean lecithin (≥97%) was supplied from Carl Roth GmbH (Karlsruhe, Germany), and silver nitrate from Gatt Koller (GmbH, Absam, Austria). Phosphate-Buffered Saline (PBS, pH 7.4) tablets, Crystal Violet, 0.5 McFarland—Remel™ McFarland Equivalence Turbidity Standards, and the necessary consumables for the antimicrobial investigations were purchased from Fisher Scientific (Loughborough, UK). Microbial strains—*Escherichia coli* ATCC 25922 and *Enterococcus faecalis* ATCC 29212 were acquired from TCS Biosciences Ltd. (Botolph Claydon, Buckingham, UK). Aqueous solutions were prepared using Millipore ultrapure water (electrical conductivity < 0.055 µS/cm, and a resistivity of 18.22 MΩ x cm at 25 °C) obtained from a Mili-Q water system (Direct-Q 3UV system). The chlorophyll extract was prepared in-house from fresh leaves of *Spinacia oleracea* L., as described in [20].

### 2.2. Botanical Synthesis of AgNPs from G. robertianum Aqueous Extract

The botanical synthesis of AgNPs was carried out in 2 stages:

#### 2.2.1. Preparation of Vegetal Extract (GR-EV)

The aqueous herbal extract from *G. robertianum* L. was obtained by Soxhlet extraction technique [21]. Briefly, an amount of 100 g of plant material (aerial parts of *G. robertianum* L.) dried and ground to the consistency of fine powder was extracted in 1200 mL solvent (ultrapure deionized water), at extraction temperature t > 80 °C, for three hours. The obtained extract was filtered at normal pressure on filter paper (Whatman no. 1, Merck KGaA, Darmstadt, Germany) and stored at 4 °C to prevent the degradation of heat-sensitive or light-sensitive phytochemicals. GR-EV extract, stored under the mentioned conditions, was subsequently used in the phytosynthesis of AgNPs.

#### 2.2.2. Phyto-Nano-Synthesis of AgNPs-GR

An appropriate volume of aqueous plant extract (GR-EV) was mixed with a 1 mM aqueous solution of AgNO_3_ in a ratio of GR-EV/AgNO_3_ solution = 1:100, *v*/*v*, in the dark, under continuous magnetic stirring, at room temperature. After 24 h, the color stabilized into a dark reddish-brown hue.

### 2.3. Preparation of Biomimetic Membranes

Soybean lecithin vesicles were used as biomimetic membrane models because phospholipids spontaneously self-assemble in aqueous media into bilayer structures which mimic fundamental amphiphilic organization of biological cell membranes, with the hydrophilic headgroups facing the aqueous environment and the hydrophobic acyl chains oriented toward the bilayer interior [22]. Thus, in the present study, the term “biomimetic” refers to the structural mimicry of the lipid-bilayer component of cellular membranes rather than to the reproduction of the full molecular complexity of a native plasma membrane. Biomimetic lipid vesicles (Lipo) were obtained by hydrating a thin film of soy lecithin with a simulant biological fluid (PBS buffer pH 7.4), according to [20]. The lipid vesicle suspensions were mechanically agitated vigorously (VIBRAX shaker—Milian USA, Columbus, OH 43230 USA, 200 rpm, ~45 min), and the obtained multilamellar lipid vesicles (MLVs) were ultrasonicated for 5 min with an ultrasonic processor (Ti MS3 probe, UP100H Hielscher Ultrasonics GmbH, 14513 Teltow, Germany). The artificial lipid bilayers were loaded with chlorophyll (Chl) acting both as an optical sensor and as a bioactive agent. Chl is known for its antioxidant, anti-obesogenic, antimutagenic, and anti-cancer activities [9]. Chlorophyll was extracted from fresh spinach leaves (*Spinacia oleracea* L.), from a 1:4 (*w*/*v*) mixture of spinach leaves: ethanol.

### 2.4. Green Bio-Design of Botanical Hybrids

**Figure 1:**
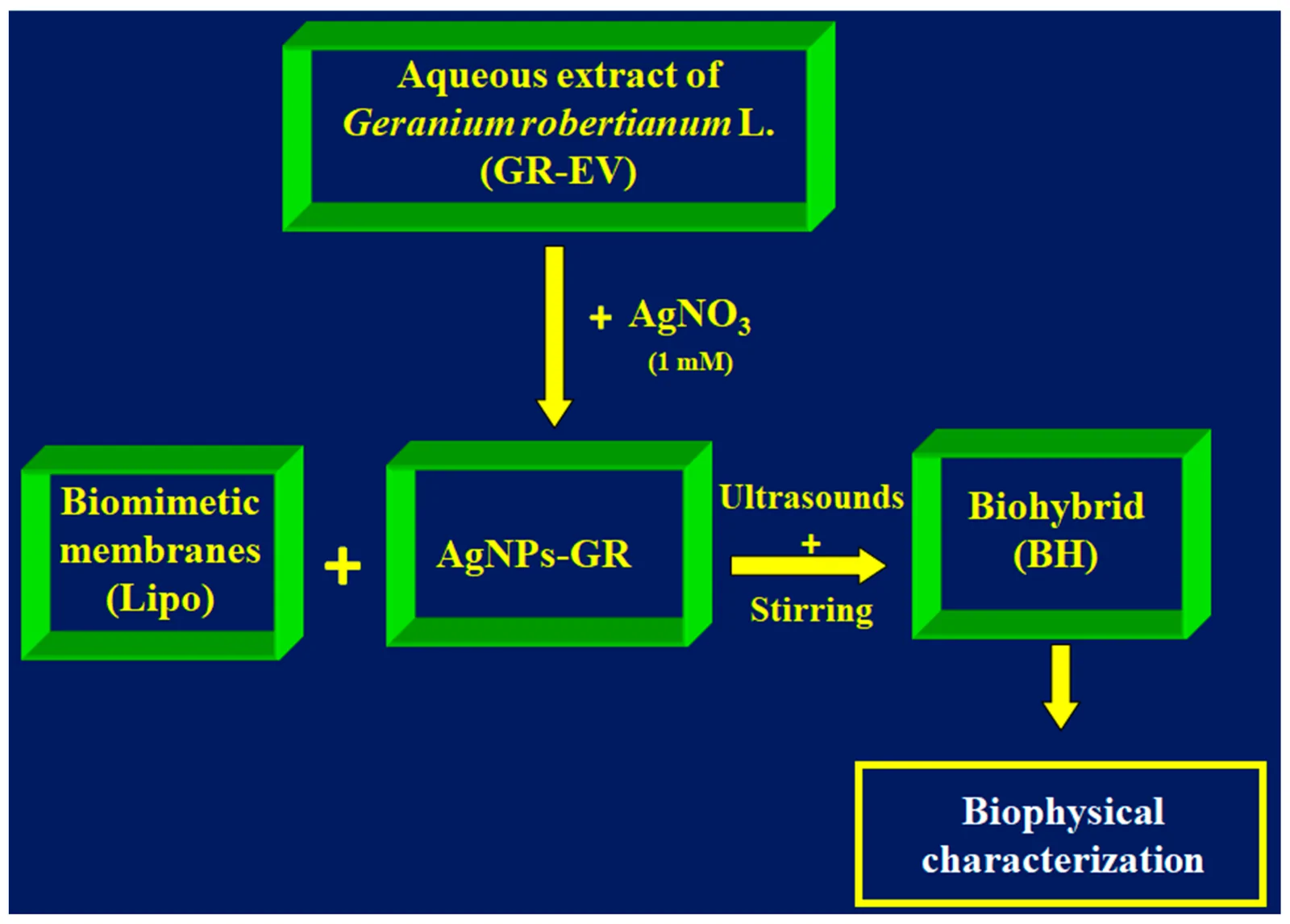
Schematic representation of the green design of biohybrids containing AgNPs phyto-generated from *G. robertianum* L.

The botanical hybrid bioentity (BH) was self-assembled from building blocks (AgNPs-GR and Lipo), by mixing appropriate volumes of Lipo and GR-AgNPs suspensions (in a ratio of 1:1, *v*/*v*), followed by strong mechanical agitation and then ultrasonication for 15 min (ultrasonic processor with Ti MS3 probe, UP100H Hielscher Ultrasonics GmbH, 14513 Teltow, Germany). The preparation of BH is schematically displayed in Figure 1. The experiments were performed in the dark to avoid photodegradation of the obtained samples.

The descriptions and abbreviations of the samples developed in this study are shown in Table 1.

### 2.5. Physicochemical and Morphological Characterization of Phyto-Derived Particles

The UV–Vis absorption spectra of the samples were recorded on a double-beam UV–Vis spectrophotometer Lambda 365+ Perkin Elmer (Perkin Elmer, Waltham, MA, USA) from 200 to 800 nm at a resolution of 1 nm.

The fluorescence emission spectra of chlorophyll-labeled samples were recorded on an LS55 Perkin Elmer fluorescence spectrometer (Perkin Elmer, Waltham, MA, USA) in the wavelength range of 600–800 nm by illuminating the samples with 430 nm excitation light. The excitation and emission slit widths were set at 6.5 and 4 nm, respectively, and the scan speed was 500 nm/min.

Fourier-transform infrared (FTIR) spectra were collected using a Perkin Elmer Spectrum 400 instrument with an attenuated total reflectance (ATR) diamond crystal (Perkin Elmer-Spectrum 400, Waltham, MA, USA) in transmittance mode. For each spectrum, scans in the 4000–650 cm^−1^ range at a spectral resolution of 4 cm^−1^ were acquired.

The average size (Zav) of prepared particles was estimated, in triplicate, by Dynamic Light Scattering (DLS), and the results were reported as the mean values ± standard deviation (SD). DLS measurements were carried out at room temperature (25 °C) on a Zetasizer Nano ZS (Malvern Instruments Inc., Worcestershire, UK), and the size distribution’s width was determined by means of the polydispersity index, PdI.

The physical stability of the samples was evaluated in terms of zeta potential (ξ, expressed in mV) measurements which were performed in ultrapure water at room temperature (25 °C) in an appropriate device of the Zetasizer Nano ZS (Malvern Instruments Ltd., Malvern, UK) by applying an electric field across the analyzed samples. The ξ measurements were carried out in triplicate, and the ξ values were reported as mean ± S.D.

The morphological characteristics of nanoparticles and extracts were examined using an ApreoS ThermoFisher (Brno, Czech Republic) scanning electron microscope (SEM) with a maximum resolution capability of 0.7 nm. Top-view SEM images of the obtained particles were captured at a working voltage of 10 kV and a pressure of 1 × 10^−3^ Pa. To minimize electrical charging during analysis, the samples were coated with a thin layer of gold. Additionally, the Energy Dispersive X-ray (EDX) detector of a FEI/Phillips scanning electron microscope (Hillsboro, OR, USA) was used to acquire the EDX spectra for all samples.

The specimens were subsequently characterized by atomic force microscopy (AFM) to obtain complementary information on particle dimensions, surface morphology, and spatial distribution. Prior to AFM imaging, silicon (Si) substrates (10 × 10 mm^2^) were thoroughly cleaned and dried. Following sonication, a 10 μL aliquot of each nanoparticle suspension was drop-cast onto the freshly cleaned Si substrates and allowed to dry overnight under ambient conditions. AFM measurements were performed in tapping mode using an A.P.E. Research A100-SGS microscope (A.P.E. Research S.r.l., Trieste, Italy) equipped with aluminum (Al)-coated Si cantilevers (NSC15/Al BS, MikroMasch OÜ, Tallinn, Estonia) having a nominal resonance frequency of 325 kHz, a nominal spring constant of 46 Nm^−1^ and a nominal tip radius below 10 nm. The acquired images were flattened and post-processed using the Gwyddion software package (version 2.52), which was also employed to extract and analyze the particle height profiles.

### 2.6. Determination of Total Phenolic Content (TPC) of Plant Extract and AgNPs

Total phenolic contents (TPC) of the *G. robertianum* L. extract were determined spectrometrically using the Folin–Ciocâlteu method [23] using gallic acid as a standard to prepare a calibration curve [24]. Briefly, an aliquot of the extract was mixed thoroughly with 5 mL Folin–Ciocâlteu reagent and 10 mL of sodium carbonate solution (7.5%). The mixture was vortexed for 15 s and allowed to stand dark for 60 min and then absorbance was measured at 760 nm using a JASCO UV-Vis V-530 spectrophotometer (Jasco International Co., Ltd., Tokyo, Japan). The total phenolic content was calculated using a gallic acid calibration curve (R^2^ = 0.9988) and the results were expressed as mg of gallic acid equivalent/g of dry extract (mg/GAE g^−1^). Samples were analyzed in triplicate.

### 2.7. In Vitro Investigation of Antioxidant Activity

The in vitro antioxidant properties of the samples were tested on short-lived free radicals (chemiluminescence technique) and long-lived free radicals (DPPH assay).

#### 2.7.1. Chemiluminescence (CL) Technique

CL technique was applied using Luminol (10^−5^ M)–H_2_O_2_ (10^−5^ M) as a generator system, in TRIS-HCl buffer pH 8.79, using Sirius Luminometer (Berthelot—GmbH Germany). This mixture (200 µL luminol, 750 µL Tris-HCl buffer solution, and 50 µL H_2_O_2_) was used as a blank. For each sample, the reaction medium contained 100 µL of sample, 200 µL of luminol, 650 µL of Tris-HCl buffer solution, and 50 µL of H_2_O_2_ [25]. In this method, a cascade of reactions with light emission occurs that results in highly reactive, short-lived free radicals (such as peroxyl or hydroxyl radicals) [26]. The antioxidant activity AA% of the samples was calculated by using the following relation:

(1) $$AA(\%)=\left(\frac{I_{0}-I_{s}}{I_{0}}\right)\times 100$$

where I_0_ = the maximum CL intensity for the reference (the blank) at t = 5 s; Is = the maximum CL for the sample at t = 5 s [24].

#### 2.7.2. DPPH Assay

DPPH method was used to quantitatively test the long-lived free radical (DPPH^•^) scavenging activity (SR%) according to [21]. A DPPH solution (80 μM) was freshly prepared in 95% methanol. A volume of 500 μL of this solution was allowed to react with 70 μL of sample and the absorbance was measured at 517 nm. The DPPH^●^ scavenging assays were analyzed in triplicate. The radical scavenging activity (SR%) was calculated as follows:

(2) $$SR\%=100\times (1-\frac{{Abs}_{sample}-{Abs}_{blank}}{{Abs}_{control}})$$

### 2.8. In Vitro Antibacterial Activity Analysis

To obtain homogeneous and representative suspensions necessary for evaluating antimicrobial activity, the samples were sonicated in an ultrasonic bath for 15 min at a controlled temperature and then vortexed before sampling. Testing was performed on the reference bacterial strains *Escherichia coli* ATCC 25922 and *Enterococcus faecalis* ATCC 29212, grown on PCA (*Plate Count Agar*) (HIMEDIA, Einhausen, Germany) at 37 °C for 18–24 h to obtain pure, viable, metabolically active cultures. For further assays, bacterial suspensions standardized to 0.5 McFarland (1.5 × 10^8^ CFU/mL) were prepared in sterile 0.9% NaCl (Sigma-Aldrich, USA).

#### 2.8.1. Qualitative Evaluation of the Antimicrobial Activity of Obtained Samples

For the preliminary evaluation of antimicrobial activity, an adapted agar diffusion method was used. The bacterial suspensions were uniformly plated on Mueller–Hinton agar (Sigma-Aldrich, USA). A volume of 5 μL of each tested solution was applied to the agar surface, and then the plates were incubated at 37 °C for 24 h. Antimicrobial activity was evaluated by measuring the diameter of the inhibition zones, expressed in mm. All determinations were performed in duplicate, and the results were reported as mean ± SD.

#### 2.8.2. Quantitative Evaluation of Antimicrobial Activity of Prepared Samples

To determine the minimum inhibitory concentration (MIC) and the minimum bactericidal concentration (MBC), the tested compounds were evaluated using the binary serial microdilution method, using TSB (Tryptic Soy Broth) (Eur. Pharm. Scharlau, Sentmenat, Spain) distributed in sterile 96-well microplates. BH and its components were two-fold diluted directly into the culture medium (*v*/*v*, 100 μL/100 μL), and all wells were inoculated with 10 μL of a standardized bacterial suspension. After incubation at 37 °C for 24 h, the MIC was evaluated macroscopically and spectrophotometrically by measuring absorbance at 620 nm using a FlexStation 3 UV–Vis spectrophotometer (Molecular Devices, LLC., San Jose, CA, USA). Microbial viability was expressed as a percentage relative to the positive (the well that contained only the bacterial suspension and the culture medium) and negative (the well that contained only culture medium) controls. To determine the MBC, 5 μL from wells without visible turbidity was spotted onto PCA medium and incubated at 37 °C for 24 h. MBC was defined as the lowest concentration at which no bacterial growth was observed on the solid medium.

#### 2.8.3. Influence of the BH on the Phenotypic Expression of Soluble Virulence Factors

The extracellular enzymatic profile was evaluated by testing for the production of pore-forming toxins and enzymes (hemolysins, lipases and lecithinases), DNase, proteases (caseinase) and glucosidases (amylase, esculinase) using specific culture media containing the substrate for each enzymatic activity, as described by Corbu et al. [27]. For this purpose, 5 μL of untreated bacterial suspensions were spotted, as well as treated cultures from the wells corresponding to the MIC/2 and MIC/4 concentrations from the MIC plates. The evaluation of the results was performed 24 and 48 h after inoculation at 37 °C. Only virulence factors to which the control showed a positive reaction are reported. The impact of the compounds on the metabolic profile was evaluated semi-quantitatively by comparing the halo area relative to colony size in treated versus untreated strains and by calculating enzymatic activity inhibition according to Marinas et al. [28].

#### 2.8.4. Modulation of Adhesion and Biofilm Formation on Inert Substrate

To investigate the potential of BH to affect bacterial biofilm formation under in vitro conditions on an inert (abiotic) substrate, the violet crystal microtiter assay was used. After the spectrophotometric reading of the MIC microplates, the wells were emptied, washed to remove the non-adhered bacteria, then the biofilm formed on the plastic walls was fixed by methanol, colored with violet crystal, resuspended with an acetic acid 33% solution and finally the absorbance of the colored suspension was measured spectrophotometrically at 490 nm [28]. The concentration at which bacterial adhesion is below 10% of the control culture value was considered the Minimum Biofilm Eradication Concentration (MBEC) which is the lowest concentration of the sample that inhibits adhesion to an inert substrate.

#### 2.8.5. Statistical Analysis

Statistical analysis of the data was performed using GraphPad Prism v10 software, and the threshold for statistical significance was set at *p* < 0.05. The experiments were independently repeated three times for each sample, and each condition was measured in technical duplicate. Technical duplicates were averaged to obtain one value per independent experiment, and the data are reported as mean ± SD calculated from the three independent experiments. The One-Way ANOVA experimental design, with multiple comparisons using the Tukey test, highlighted the antimicrobial activity of the phytosynthesized silver nanoparticles, while the Two-Way ANOVA experimental design, with multiple comparisons using the Sidak test, highlighted the modulation of the production of soluble virulence factors in the presence of variable concentrations (MIC/2 and MIC/4) of the analyzed compounds.

## 3. Results

### 3.1. Optical Characterization of Botanical Entities

The botanical-derived samples were optically analyzed by UV–Vis absorption, fluorescence emission, and FTIR–ATR spectroscopy, in order to get deep insights into the phytosynthesis of Ag NPs and the BH formation.

**Figure 2:**
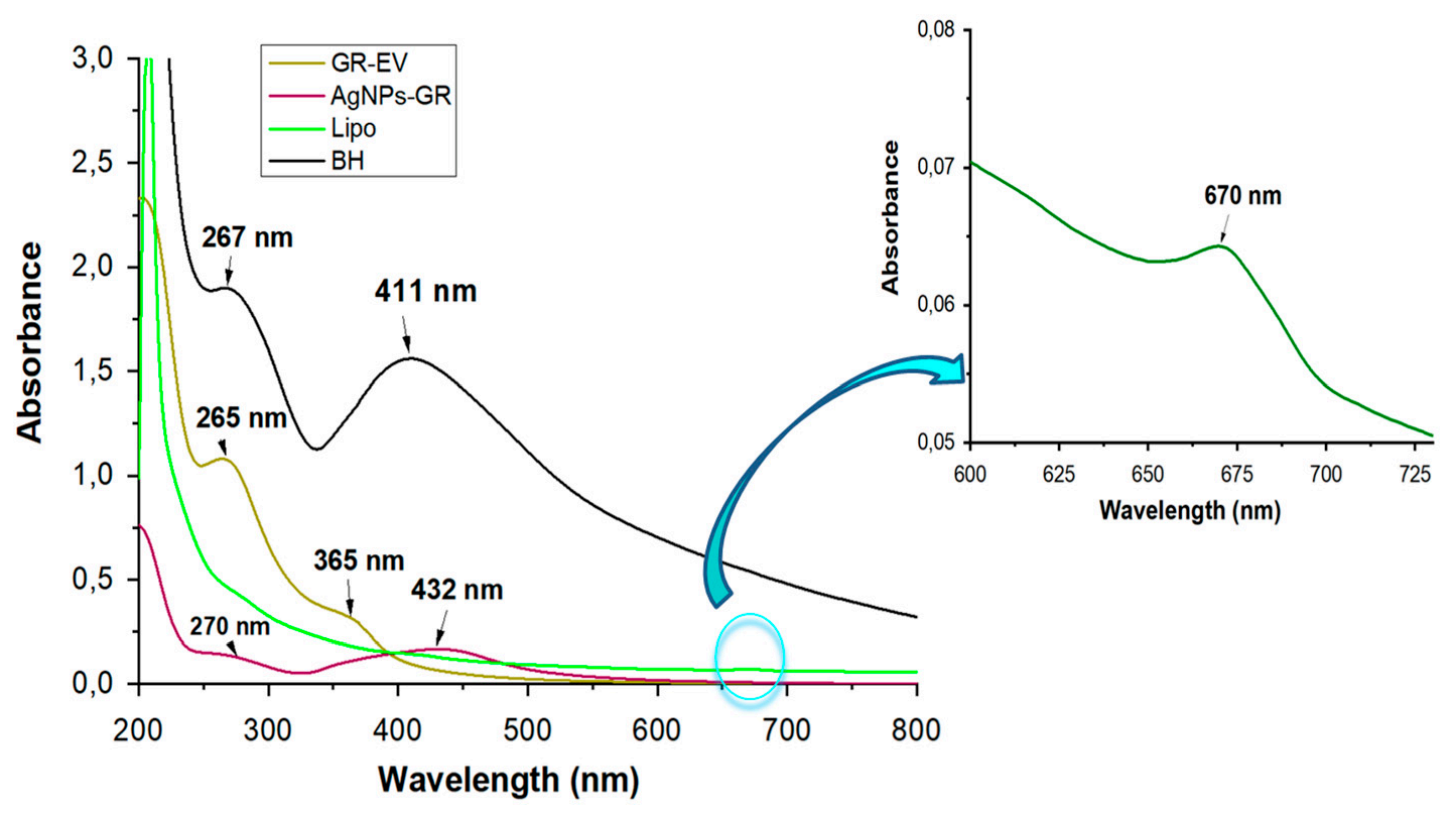
UV–Vis absorption spectra of samples derived from *G. robertianum* (GR-EV and AgNPs-GR), biomimetic membranes (Lipo) and the BH. Inset: The spectral fingerprint of Chl in liposomes.

The overlaid UV–Vis absorption spectra (Figure 2) of all samples revealed the spectral signature of AgNPs-GR in silver particles’ suspension and in BH.

**Figure 3:**
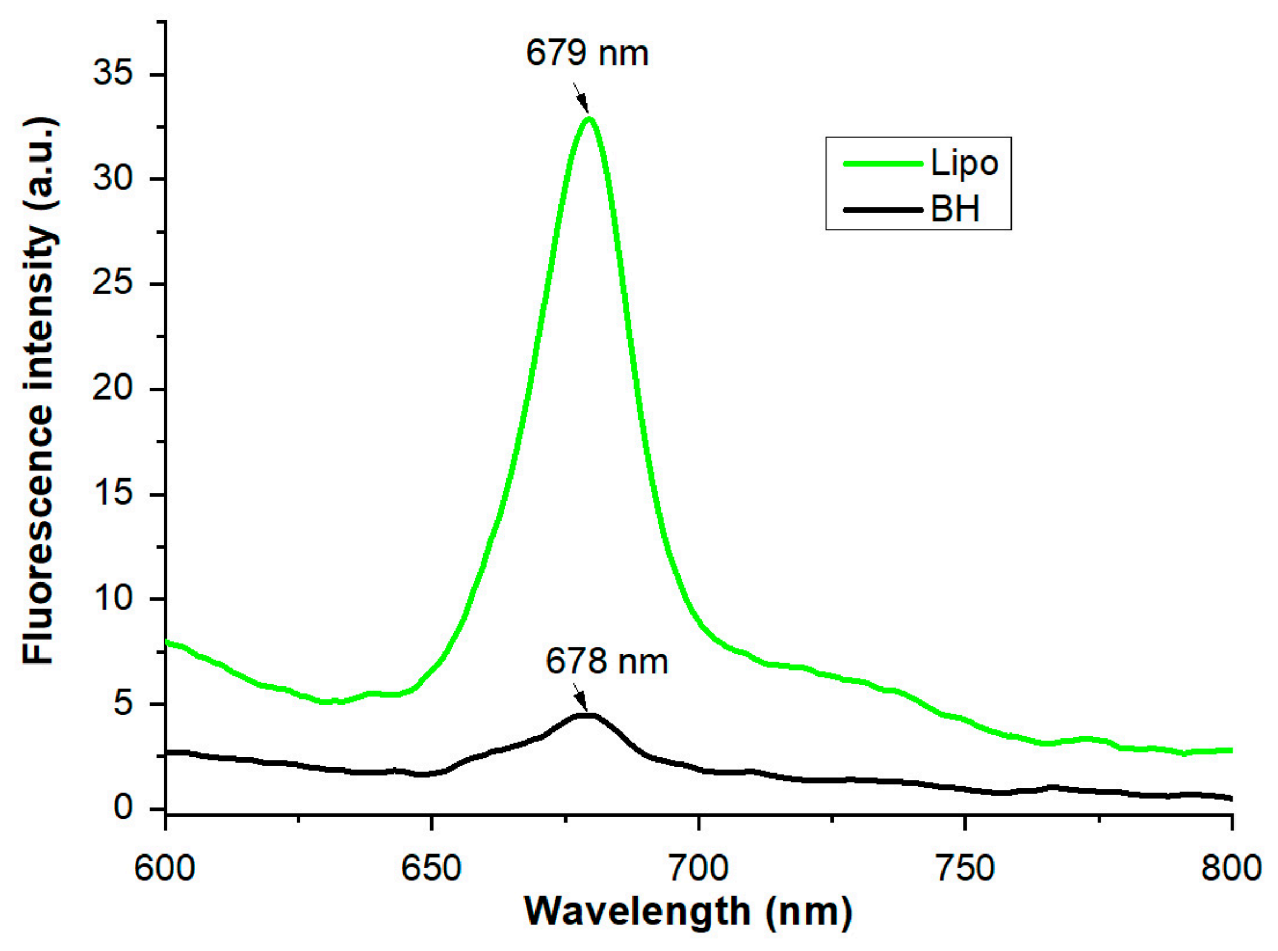
Fluorescence emission spectra of chlorophyll-labeled bio-entities (λ_exc_ = 430 nm).

Fluorescence spectroscopy showed that chlorophyll embedded in the biomimetic membranes exhibited an emission maximum at 679 nm in Lipo and at 678 nm in BH upon excitation at 430 nm (Figure 3). A pronounced decrease in chlorophyll fluorescence intensity was observed in BH compared with Lipo.

**Figure 4:**
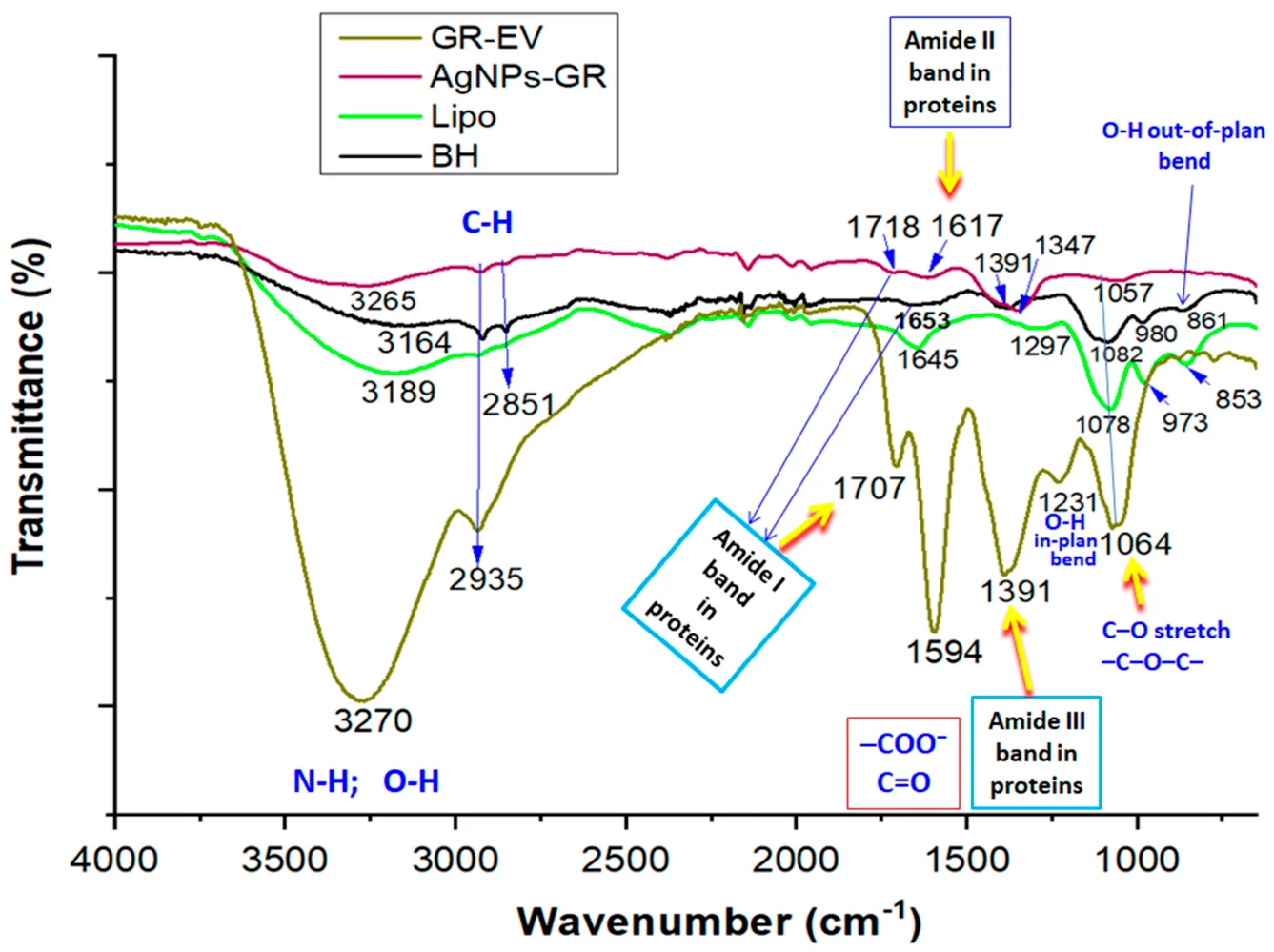
Comparative presentation of FT-IR ATR spectra of all the obtained samples.

The FT-IR ATR spectra allow identification of the functional groups that could be involved in the AgNPs-GR phytosynthesis, and also in the BH formation (Figure 4).

### 3.2. Evaluation of Physical Stability of Developed Particles

**Figure 5:**
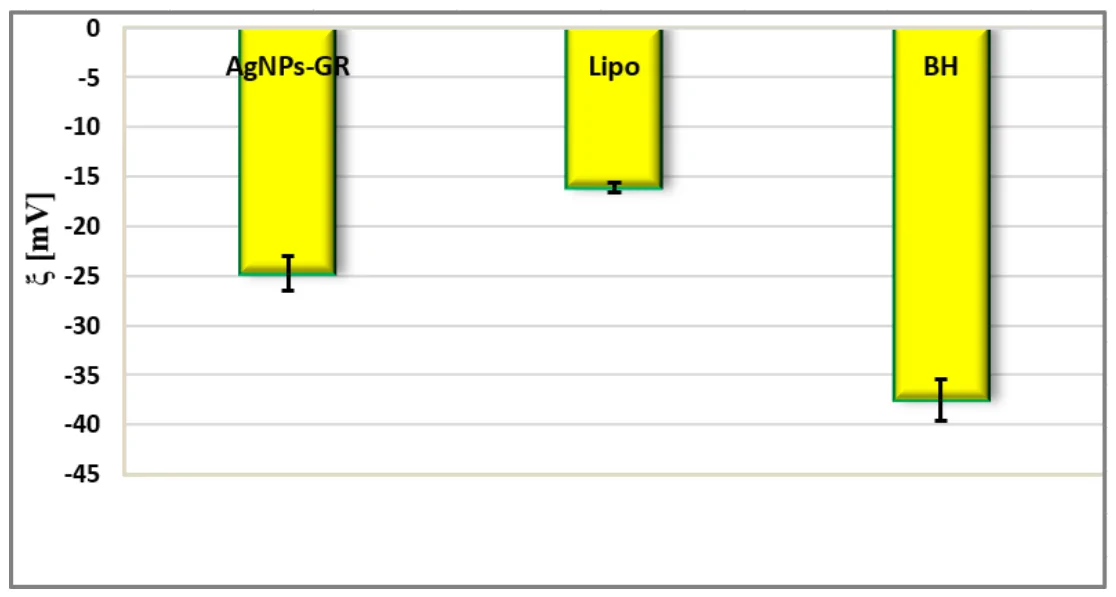
The electrokinetic potential values of the phyto-derived particles.

The electrical charge of phyto-developed particles, an indicator of the physical stability of colloidal systems, was quantified through zeta potential (ξ, expressed in mV) measurements (Figure 5) *via* electrophoretic mobility of the particles in suspensions subjected to an electric field [29].

### 3.3. Size Estimation, Morphological and Compositional Analysis of Samples

**Figure 6:**
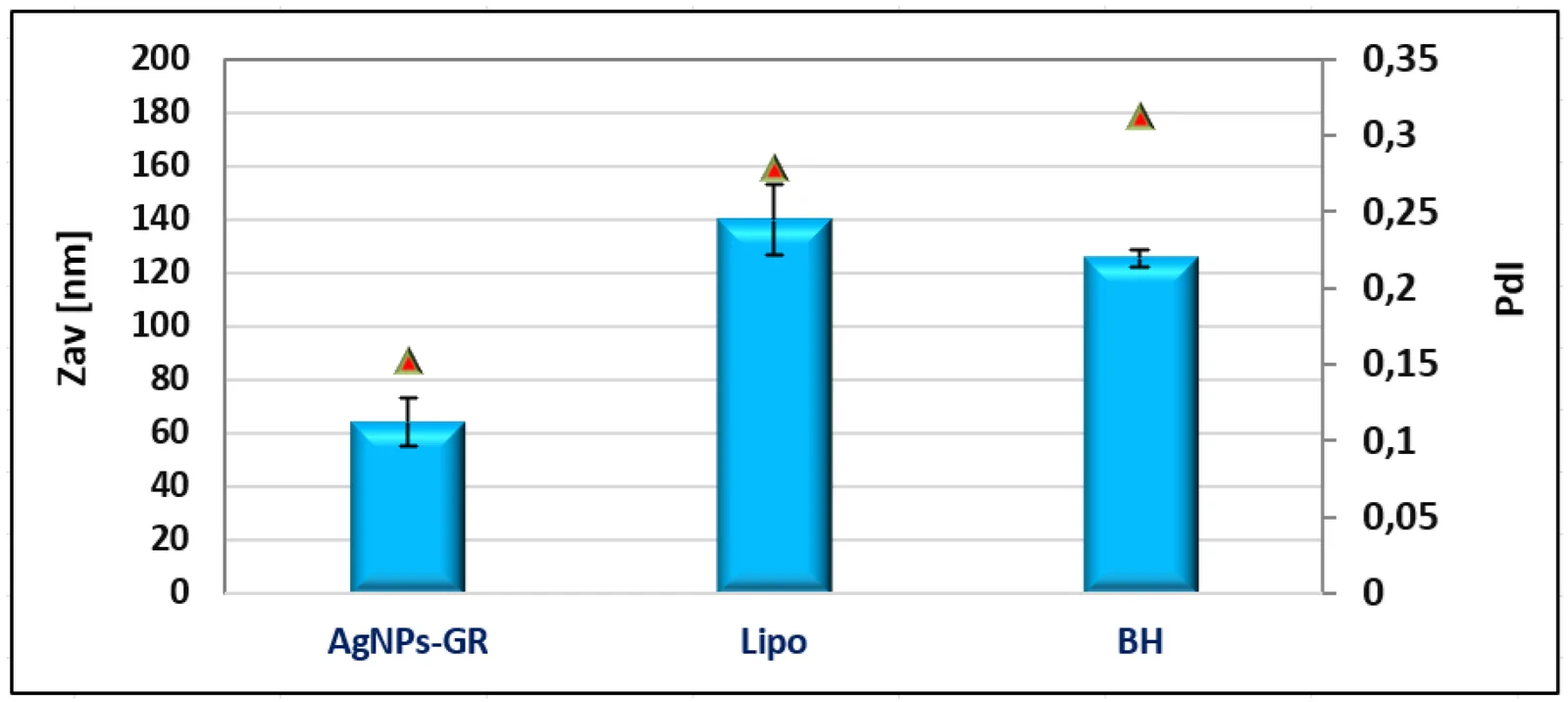
Average particle size (Zav, nm) and polydispersity index (PdI) of obtained particles, estimated by dynamic light scattering (DLS) measurements. Experiments were carried out in triplicate, and the data are expressed as mean ± SD.

DLS analysis (Figure 6) of BH revealed an average hydrodynamic diameter of 125.40 ± 3.15 nm and a PdI of approximately 0.31. The BH population was therefore smaller on average than Lipo (~140 nm) but larger than AgNPs-GR (~64 nm).

**Figure 7:**
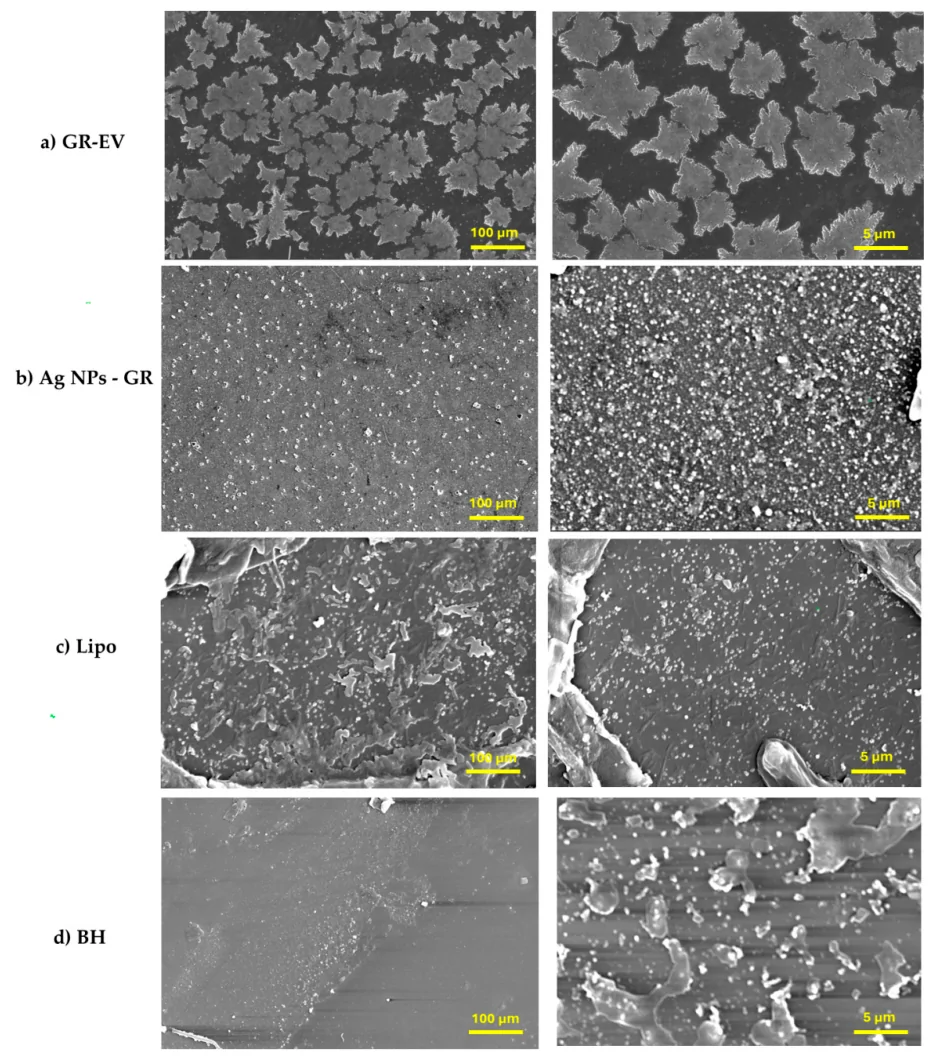
SEM images of (**a**) GR-EV, (**b**) Ag NPs-GR, (**c**) Lipo, and (**d**) BH acquired at different magnifications.

SEM analysis (Figure 7) revealed distinct morphologies among the investigated samples. GR-EV exhibited extended irregular domains with branched, dendritic/rosette-like contours and crenulated edges, whereas AgNPs-GR showed predominantly quasi-spherical nanoscale structures. Lipo displayed vesicular and membrane-like structures distributed over a continuous phospholipid matrix, with characteristic folds and wrinkles. In contrast, BH exhibited a more heterogeneous and textured morphology, with membrane-like regions coexisting with dispersed globular features, nanoscale protrusions, and particle-associated domains.

**Figure 8:**
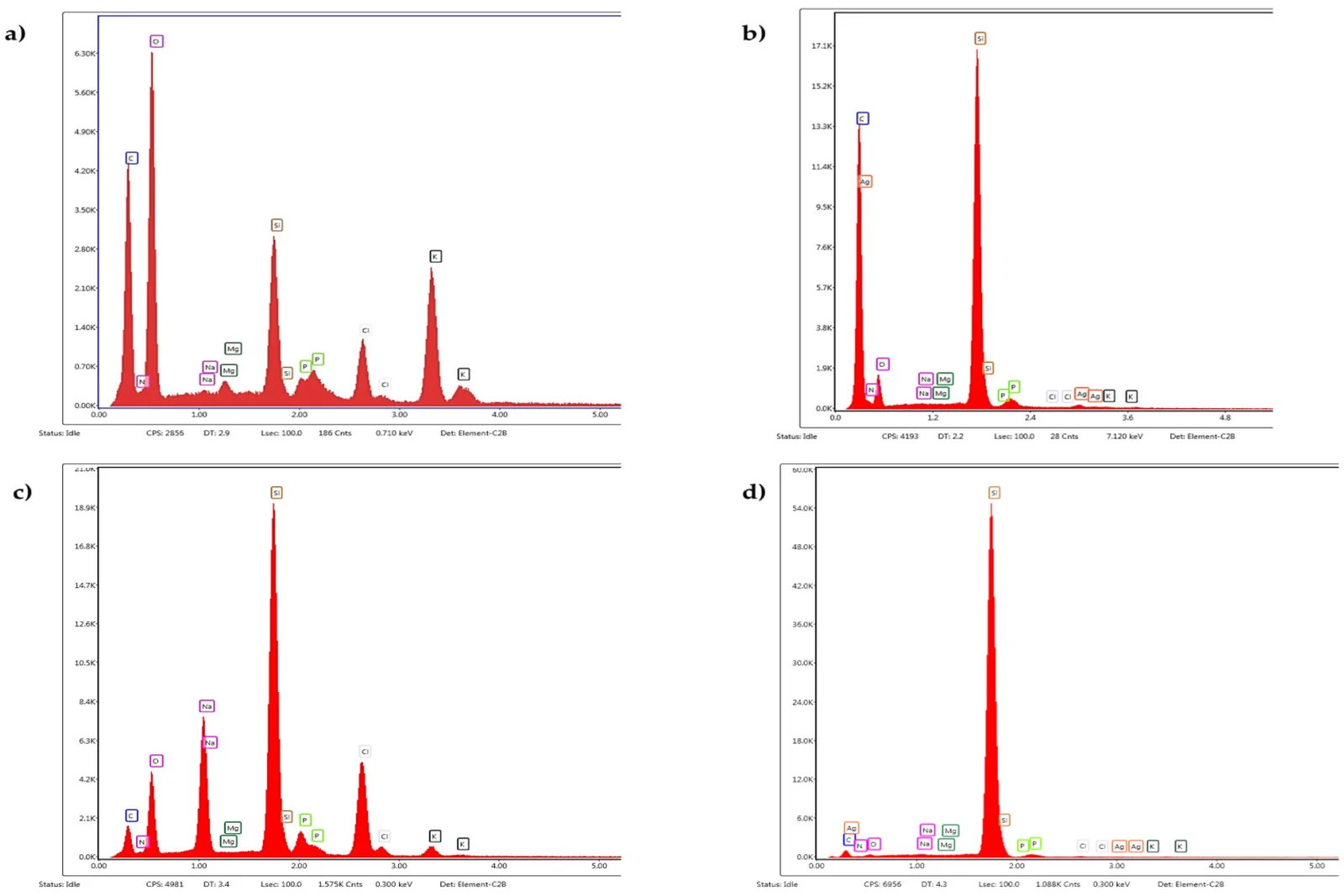
Representative EDX spectra of (**a**) GR-EV, (**b**) AgNPs-GR, (**c**) Lipo and (**d**) BH.

EDX analysis revealed distinct elemental profiles for the investigated samples (Figure 8; Table S2—Supplementary Materials).

GR-EV was dominated by C (40.03 ± 0.47 at.%) and O (44.67 ± 0.47 at.%), with lower amounts of N, Na, Mg, P, Cl, and K, while no Ag was detected. AgNPs-GR contained C (46.60 ± 3.24 at.%) and O (10.29 ± 2.59 at.%), together with a Ag signal in the ~3 keV region corresponding to 0.30 ± 0.33 at.%; N, Na, Mg, P, Cl, and K were also detected at lower levels.

Lipo contained C (16.24 ± 8.03 at.%) and O (30.75 ± 2.62 at.%), together with P (7.45 ± 2.46 at.%), Na (18.86 ± 3.43 at.%), Cl (3.65 ± 1.93 at.%), K (1.11 ± 0.32 at.%), Mg (0.44 ± 0.06 at.%), and N (0.43 ± 0.20 at.%). No Ag signal was detected.

BH contained C (35.89 ± 3.26 at.%), N (2.85 ± 1.22 at.%), O (23.18 ± 6.80 at.%), P (5.29 ± 2.55 at.%), and Ag (0.13 ± 0.059 at.%), together with Na, Mg, Cl, and K. The Ag signal was observed in the ~3 keV region, whereas P was detected in the phospholipid-associated region.

**Figure 9:**
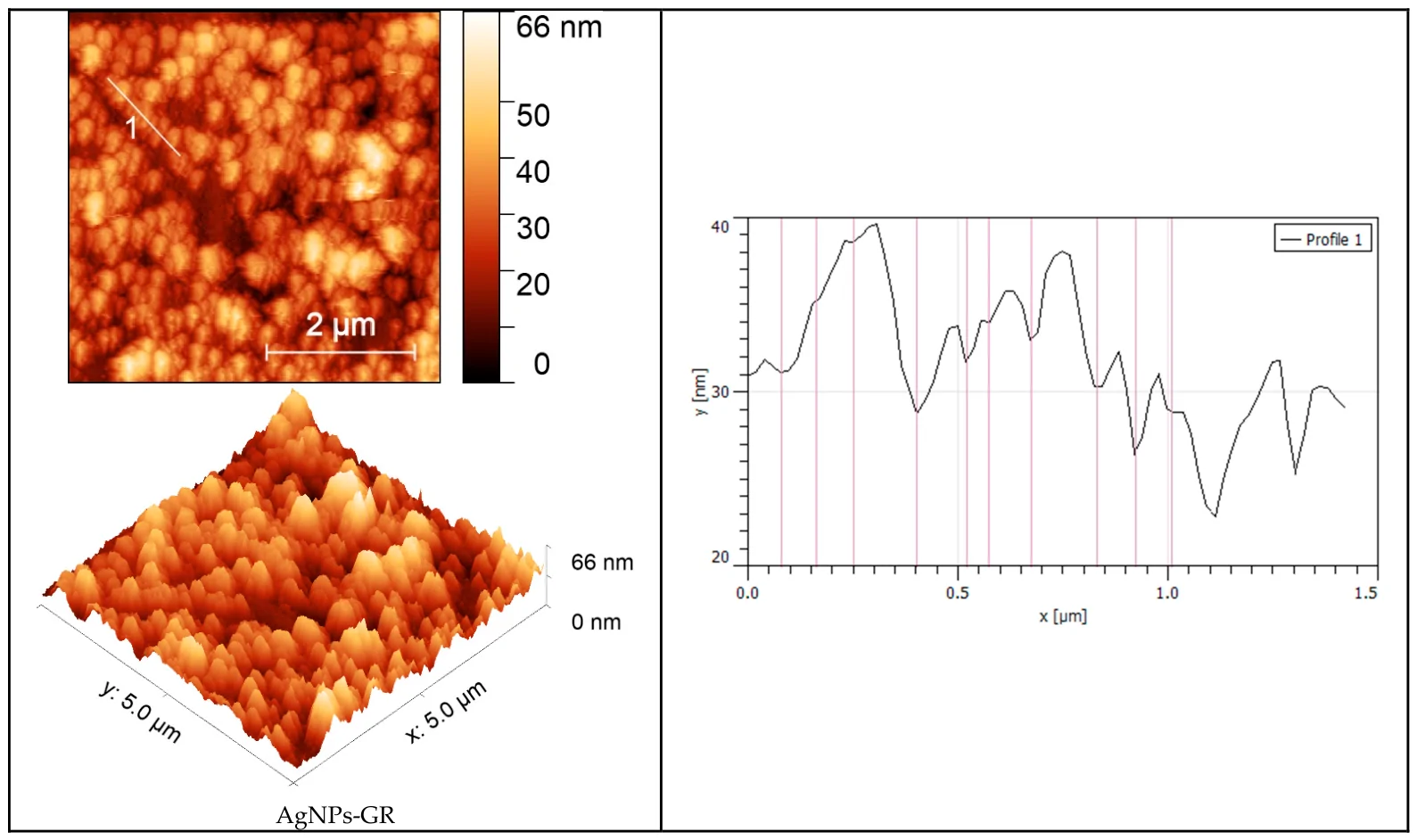

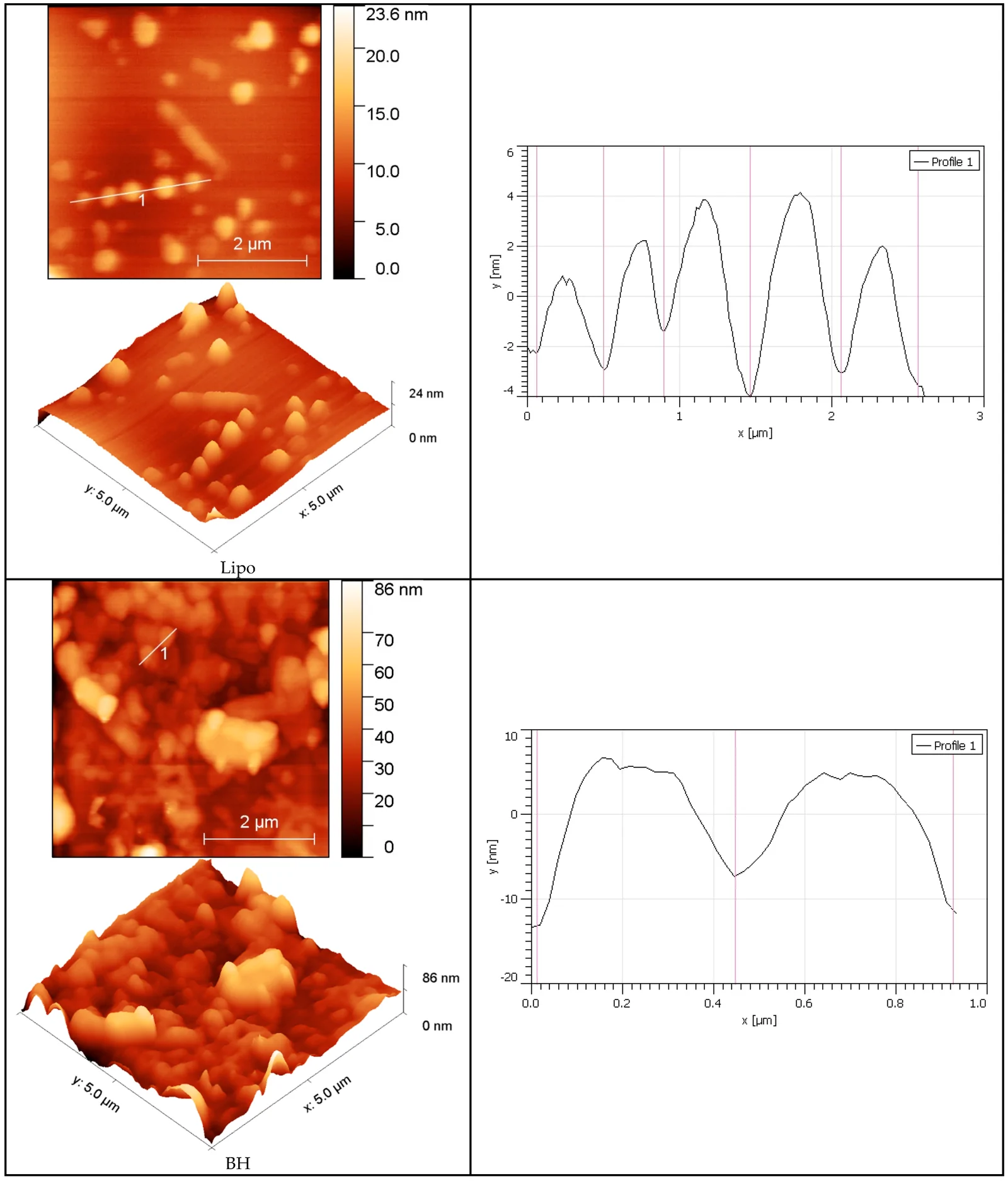
AFM analysis of the obtained particles.

Surface topography of all samples was investigated by AFM analysis (Figure 9) and revealed (quasi-)spherically shaped nano-entities. The RMS roughness values were 9.52 nm for AgNPs-GR, 1.939 nm for Lipo, and 10.69 nm for BH (Table 2), with BH exhibiting the highest surface roughness.

The specific chemical elements in the obtained samples were identified by EDX (Figure 8).

The value of root mean square (RMS) roughness for AgNPs-GR was 9.52 nm (Table 2).

### 3.4. Estimation of Total Phenolic Content for G. robertianum Extract and AgNPs-GR

The aqueous extract of *G. robertianum* presented high phenolic content TPC = 89.35 ± 0.17 mg GAE g^−1^, while AgNPs-GR showed a value of 9.17 ± 0.13 mg GAE g^−1^ for TPC. This sharp drop in TPC value highlights that 89.73% of the polyphenol amount was used in the phyto-synthesis of AgNPs-GR.

### 3.5. Investigation of Biological Performance of BH

#### 3.5.1. Antioxidant Activity Evaluation

**Figure 10:**
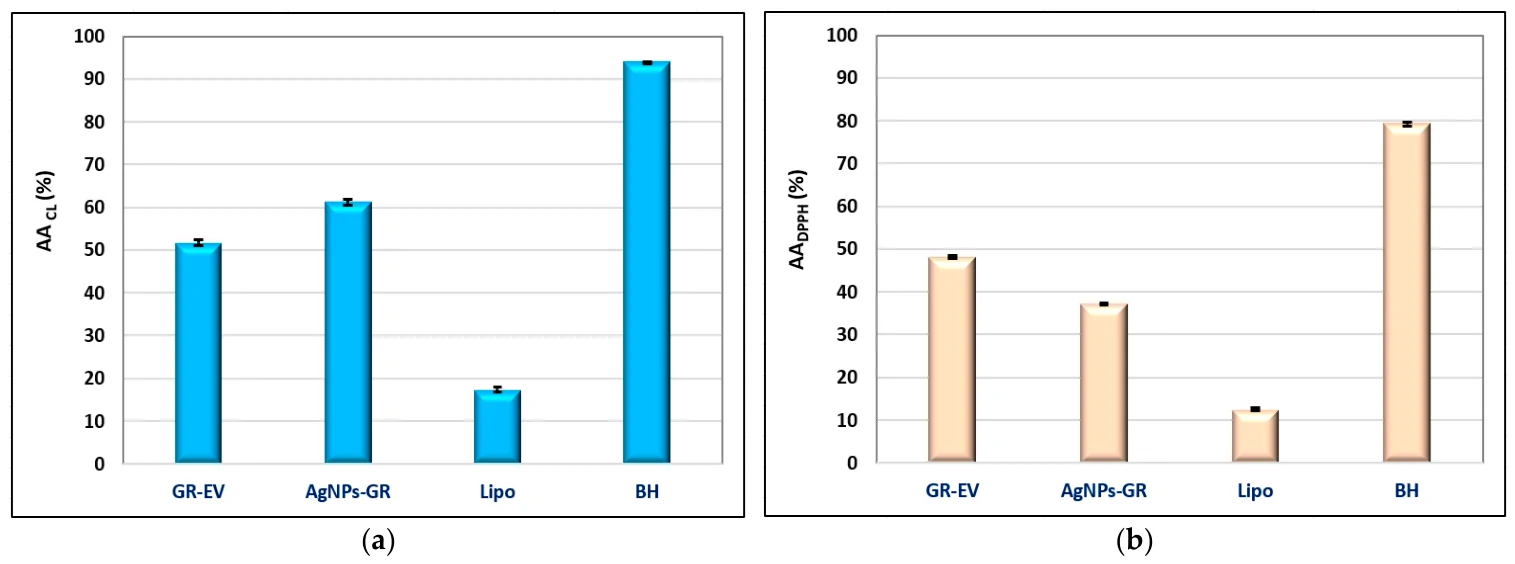
Antioxidant profile of the obtained samples estimated using the CL technique (**a**) and DPPH assay (**b**).

The antioxidant activities of GR-EV, AgNPs-GR, Lipo, and BH determined by CL and DPPH assays are presented in Figure 10. Lipo exhibited the lowest antioxidant response among the investigated samples, whereas GR-EV showed measurable activity in both assays. AgNPs-GR exhibited an antioxidant activity of AA_CL_ = 61.19% in the CL assay and AA_DPPH_ = 37.23% in the DPPH assay. BH presented the highest AA% values against both short-lived free radicals (AA_CL_ = 93.84%) and long-lived free radicals (AA_DPPH_ = 79.22%).

#### 3.5.2. Antibacterial Activity Evaluation

The antibacterial activity of the investigated samples was evaluated by agar diffusion assay [30] and MIC, MBC, and MBEC assays. For direct comparison, the individual building blocks were tested at composition-matched amounts corresponding to their contribution to the BH formulation. In the agar-diffusion assay, GR-EV and Lipo produced no detectable inhibition zones against either bacterial strain, whereas AgNPs-GR produced inhibition zones of 6.50 ± 0.71 mm against *E. faecalis* and 5.50 ± 0.71 mm against *E. coli*. BH produced inhibition zones of 10.00 ± 0.00 mm against both strains (Table 3). The AgNPs-GR sample was diluted two-fold to match its concentration in BH.

In the broth-based assays, BH and AgNPs-GR exhibited identical MIC, MBC, and MBEC (Table 4) values against *E. coli* (125 µL/mL). Against *E. faecalis*, both formulations showed an MIC of 250 µL/mL and an MBEC of 250 µL/mL, whereas BH exhibited an MBC of 250 µL/mL and AgNPs-GR an MBC > 250 µL/mL. Thus, the difference between BH and AgNPs-GR was more pronounced in the agar-diffusion assay than in the quantitative broth assays.

Regarding virulence-factor modulation, the tested formulations produced strain-, concentration-, and time-dependent effects. For *E. faecalis*, several samples reduced haemolysin production after 24 h, whereas the effect was not maintained in all cases after 48 h. GR-EV inhibited esculinase at MIC/2, while at MIC/4 all tested samples showed inhibition after 48 h. For *E. coli*, Lipo and BH inhibited amylase production after 48 h at MIC/4. In some conditions, increased production of selected soluble enzymes was also observed.

## 4. Discussion

### 4.1. Physicochemical and Morphological Characterization of Phyto-Derived Particles

The present study examines the formation and properties of the BH system based on the integration of phyto-derived AgNPs-GR with chlorophyll-containing biomimetic phospholipid membranes.

The phytonanosynthesis of AgNPs-GR was evidenced by the appearance of a single SPR band at 432 nm, characteristic of spherical AgNPs (Figure 2) [31]. As we can see, the spectrum of the plant extract GR does not show this band. The spectral fingerprint of AgNPs-GR was retained in the BH spectrum, with a shift from 432 nm to 411 nm. This shift indicates a modification of the local environment of the silver nanostructures following their association with the biomimetic membrane component. In addition, the UV absorption bands associated with plant-derived components exhibited small shifts after AgNP formation and subsequent incorporation into BH. These optical changes are consistent with interactions between the parent components during assembly of BH, in line with our previous report [20]. The UV–Vis absorption spectrum of Lipo contains the chlorophyll spectral signature at 670 nm (inset of Figure 2). In the UV–Vis absorption spectrum of GR-EV extract, the spectral signature of polyphenolic compounds is observed (365 nm), as is that of the aromatic amino acid residues of proteins (265 nm) [32]. The last UV absorption band of 265 nm within the GR-EV spectrum shifted to 270 nm in AgNPs-GR, and to 267 nm in BH. These spectral shifts support the formation of AgNPs-GR and the subsequent assembly of the BH system, in agreement with our previous report [20].

The pronounced fluorescence quenching observed for chlorophyll in BH suggests close interaction between the chlorophyll-containing phospholipid structures and AgNPs-GR (Figure 3). Such quenching may involve energy and/or electron transfer processes associated with the proximity of the two components. These findings are consistent with previous reports [20].

FT-IR ATR band assignment for the obtained samples is displayed in Table S1 (Supplementary Materials).

After the addition of AgNO_3_ solution to the GR-EV aqueous extract, some spectral changes occurred, as follows:

- The intense and sharp band at 3270 cm^−1^ in GR-EV, attributed to hydrogen-bonded O–H (bending and stretching vibrations of hydroxyl groups in alcohols, and phenolic compounds) and N–H stretching vibrations of amines or amides [33], shifted to 3265 cm^−1^, while weakened and broadened in AgNPs-GR.
- The band at 1707 cm^−1^ in GR-EV, assigned to Amide I, due to carbonyl stretch in proteins; carboxylate groups (–COO^−^); N–H bend [24], shifted to 1718 cm^−1^ in AgNPs-GR.
- The strong and sharp band at 1594 cm^−1^ in GR-EV, assigned to Amide II due to N–H bending and C–N stretching in proteins [24], weakened, broadened, and shifted to 1617 cm^−1^ in AgNPs-GR [34].
- The band at 1231 cm^−1^ in GR-EV (due to the contribution of primary or secondary O–H in-plane bends, aromatic ethers, aryl–O stretch, aromatic primary amine, or C–N stretch [24]) disappeared in AgNPs-GR.
- The sharp and intense band at 1064 cm^−1^ in GR-EV, assigned to C–O stretch; –C–O–C– ether; antisymmetric stretching of –C–O group of polysaccharides and/or chlorophyll [24], shifted to 1057 cm^−1^ and weakened in AgNPs-GR.

These spectral changes support the involvement of plant-derived functional groups in the reduction and stabilization processes associated with AgNPs-GR formation. As observed in Figure 4 and in Table S1 (Supplementary Materials), the FT-IR ATR spectrum of AgNPs-GR displays residual peaks from phyto-derived capping agents, such as O–H, –COO^−^; –C–O–C–, C–N and N–H, which offer bioactivities to AgNPs-GR as further demonstrated.

Lipo spectrum revealed an intense, broad band centered at 3189 cm^−1^ (assigned to the O−H stretching in water molecules tightly associated with the biomimetic membranes *via* hydrogen bonds [35]) that shifts to 3164 cm^−1^ in BH. Other peaks presented in Lipo FTIR ATR spectrum are located at 2935 cm^−1^ (C–H anti-symmetric stretching vibration in the CH_2_ groups of alkyl chains of lipids [33]), 2851 cm^−1^ (C–H symmetrical stretch vibration of alkyl chains [33]), 1645 cm^−1^ (stretching vibrations of the ester carbonyl groups of lipids [35]), 1297 cm^−1^ (antisymmetric PO_2_^−^ stretching in lipids [35]), 1078 cm^−1^ (symmetric PO_2_^−^ stretching in lipids; antisymmetric stretching of –C–O group of chlorophyll [33]), 973 cm^−1^ (antisymmetric choline group (N^+^−(CH_3_)_3_) stretching vibrations in lipids [33]), and 853 cm^−1^ (stretching vibrations of –CH_2_– in lipids [33]).

In the BH spectrum, the bands located at 2935 cm^−1^ (C–H anti-symmetric stretching vibration in the CH_2_ groups of alkyl chains of lipids) and 2851 cm^−1^ (C–H symmetrical stretch vibration of alkyl chains [33]), became sharp and more intense as compared to the spectra of the components Lipo and AgNPs-GR. The bands located at 973 cm^−1^ and at 853 cm^−1^ in the Lipo spectrum weakened and shifted to 980 cm^−1^ and at 861 cm^−1^ in the BH spectrum. Moreover, the band at 1347 cm^−1^ (carboxylates; amide III band in proteins [33]) in AgNPs-GR weakened and shifted to 1391 cm^−1^ in the BH spectrum.

These spectral modifications suggest the involvement of O–H, –COO^−^, C–O–C, and N-containing functional groups in interactions occurring during BH assembly. Because BH is formed by supramolecular association rather than by synthesis of a new covalently bonded compound, the observed changes are expected mainly as shifts and modifications of existing bands rather than the appearance of entirely new FTIR bands [36].

Taken together, the UV–Vis, fluorescence, and FTIR results indicate changes in both the optical environment and the local chemical environment of the parent components following BH assembly.

Empty biomimetic lipid vesicles showed poor stability (ξ_Lipo_ = −16.5 mV), while AgNPs-GR exhibited good stability (ξ = −24.8 mV) due to the capping geranium-derived molecules bearing negatively charged functional groups (Figure 5). As shown by FT-IR ATR analysis, there are molecules containing hydroxyl, carbonyl, and carboxyl groups on the surface of developed AgNPs-GR that ensure colloidal stability and biological activities. Eker et al. highlighted the key role of plant-based *green* synthesis in enhancing the AgNPs stability, by using natural phyto-molecules as both capping and stabilizing agents in the AgNPs formation [37].

The zeta potential value of AgNPs-GR is in line with other zeta potential values for phytogenic AgNPs derived from leaf extracts reported in the scientific literature. For instance, Sharifi-Rad et al. prepared AgNPs from *Lallemantia Royleana* leaf extract with a zeta potential value of −24.1 mV [38]. Keskin et al. [39] obtained AgNPs phytosynthesized from aqueous extract of *Anchusa officinalis* leaves, with a zeta potential value of −21.2 mV.

Following the combination of Lipo and AgNPs-GR, BH exhibited a higher absolute zeta-potential value (−37.5 mV) than either parent component. This change indicates a distinct surface-charge environment after assembly and is consistent with increased electrostatic stabilization of the resulting hybrid dispersion. These results are in line with our previous studies [40].

Together with the changes in ζ-potential and the complementary spectroscopic and compositional data, the altered hydrodynamic behavior is consistent with reorganization of the parent components following BH assembly (Figure 6). The somewhat higher PdI of BH reflects a broader particle-size distribution, consistent with the heterogeneity expected for a multicomponent self-assembled system [41].

The altered hydrodynamic size and surface charge characteristics of BH further indicate that assembly modifies the colloidal organization of the parent systems.

SEM examination of the precursor GR-EV revealed a markedly different morphology from that observed after AgNP formation. The dried plant extract formed extended irregular domains with strongly branched, dendritic/rosette-like contours and crenulated edges (Figure 7a). Their morphology is most likely associated with aggregation, crystallization, and spatial redistribution of soluble extract components during solvent evaporation. In contrast, SEM analysis of AgNPs-GR (Figure 7b) revealed predominantly quasi-spherical nanoscale structures distributed over the substrate. The latter are most likely related to particle association during drying rather than to the primary particle population in suspension. This interpretation is supported by the DLS results, which showed an average hydrodynamic diameter of 64.33 ± 9.11 nm and a low polydispersity index (PdI ≈ 0.15), indicating a relatively narrow particle-size distribution in the hydrated state. The pronounced morphological transition from the micrometric dried domains of GR-EV to predominantly quasi-spherical nanoscale structures after reaction with AgNO_3_ provides additional morphological evidence of the transformations occurring during phytosynthesis.

Similar quasi-spherical morphologies have been reported for green-synthesized AgNPs obtained from botanical extracts. Krishnakumar et al. reported spherical AgNPs with dimensions ranging from 141 to 202 nm synthesized using *Hydnocarpus pentandra* leaf extract [42], and Hashemi et al. described spherical AgNPs obtained from *Feijoa sellowiana* leaf extract exhibiting good colloidal stability and biological activity [43]. Plant-derived polyphenols, flavonoids, proteins, and other phytoconstituents may participate in the reduction of Ag^+^ ions and subsequently remain associated with the nanoparticle surface as capping and stabilizing species. Similar phytochemical-mediated stabilization has been reported for AgNPs obtained using medicinal plant extracts [44].

SEM analysis of Lipo revealed vesicular structures distributed over a continuous phospholipid matrix (Figure 7c). The surface exhibited characteristic folds and wrinkles associated with dehydration and partial collapse of phospholipid bilayers during SEM sample preparation. Such features are consistent with the intrinsic self-assembly behavior of phospholipids, which spontaneously organize into bilayer vesicles and membrane-like architectures in aqueous environments. Upon dehydration, these supramolecular assemblies may undergo structural rearrangement, deformation, association, and partial collapse, producing flattened or film-like morphologies distinct from their organization in the hydrated state. Similar behavior has been reported for lecithin-based liposomal systems and phospholipid vesicles generated through self-assembly processes [45]. Importantly, dehydration has been shown to induce morphological changes and coalescence in liposomal systems, while electron microscopy sample preparation can influence the morphology of soft lipid assemblies [46]. SEM was therefore used here to assess the morphology and surface organization of the deposited lipid assemblies, while their colloidal characteristics in suspension were independently evaluated by DLS. DLS analysis showed an average hydrodynamic diameter of 139.70 ± 13.12 nm for Lipo, with a PdI of ~0.28, indicating a moderately broad vesicle-size distribution.

The BH sample (Figure 7d) exhibited a more heterogeneous and textured morphology than either AgNPs-GR or Lipo alone. Membrane-like regions coexisted with dispersed globular features, nanoscale protrusions, and particle-associated domains, producing a surface organization distinct from those of the individual parent components. This morphological reorganization is consistent with interactions between the phyto-derived AgNPs and phospholipid assemblies during BH formation. The integration of geranium-derived metallic nanoparticles with phospholipid vesicles has previously been demonstrated by Bărbînță-Pătrașcu et al., who incorporated gold and Ag NPs green-synthesized using geranium leaf extract into phosphatidylcholine-based lipid vesicles, obtaining stable BH systems with biological activity [47]. Related structural organization has also been reported for BH combining biomimetic membranes with phyto-derived Ag/AgCl nanoparticles [40]. As for Lipo, the morphology observed after deposition and dehydration reflects the organization of the dried multicomponent assembly.

The RMS roughness obtained for AgNPs-GR was comparable to values previously reported for green-synthesized AgNPs; for example, Alahmad et al. reported an RMS value of approximately 10 nm for AgNPs synthesized using a *Hypericum perforatum* aqueous extract [48]. The higher RMS value observed for BH compared with AgNPs-GR and Lipo indicates a more pronounced nanoscale surface heterogeneity following BH assembly. Such differences in surface topography may influence the interaction of these bio-based systems with their surrounding biological environment.

The more heterogeneous morphology observed by SEM is consistent with the higher nanoscale surface roughness measured by AFM for BH, indicating a more complex surface organization than that in the individual parent components.

The EDX results further support the compositional evolution from the individual building blocks to BH. The predominance of C and O in GR-EV is consistent with the organic nature of the plant extract, while the minor elements may originate from naturally occurring organic and mineral constituents. The appearance of Ag in AgNPs-GR after reaction with AgNO_3_ provides elemental evidence for the formation of the Ag-containing nanostructures, while the accompanying plant-associated elements are consistent with the presence of phytochemical-derived surface material. Similar elemental profiles have been reported for plant-mediated AgNPs [38].

For Lipo, the presence of P is consistent with phosphate-containing phospholipid headgroups, whereas Na, Cl, and K may originate from PBS used during preparation and Mg may be associated with the incorporated chlorophyll. Importantly, BH contains both Ag and phospholipid-associated P. Their simultaneous detection, together with the organic-associated elements, supports the contribution of both AgNPs-GR and Lipo to the final hybrid formulation.

The EDX detection of both Ag and phospholipid-associated P complements the FTIR evidence of contributions from both the phytochemical/AgNP and lipid components, supporting their coexistence within the BH formulation.

The marked decrease in TPC after AgNPs-GR synthesis is consistent with the involvement of phenolic compounds in the reduction of Ag^+^ ions and in the subsequent stabilization of the formed nanoparticles. Residual phytochemicals, including unreacted polyphenols and proteins, may remain associated with the AgNP surface and contribute to its capping and stabilization. This interpretation is also supported by the UV–Vis and FTIR results and is consistent with previous reports [15].

Because a separately synthesized bare or chemically stabilized AgNP control was not included, the present experimental design does not allow the biological contribution of the metallic Ag core to be distinguished from that of the associated phytochemical capping layer. Therefore, AgNPs-GR are considered throughout this study as the complete phytosynthesized silver-containing nanostructure.

Taken together, the SEM, DLS, AFM, and EDX results demonstrate a clear progression from the individual parent architectures toward a structurally integrated BH system. The morphological evolution observed from AgNPs-GR and Lipo to BH confirms the successful self-assembly of phyto-derived silver nanostructures within a biomimetic membrane platform, yielding a hierarchical BH architecture with potential advantages for antimicrobial and biointerface-related applications. Moreover, the size of BH belonging to the range of 100–200 nm makes it suitable for biomedical applications as a bioactive carrier. In addition, the more negative zeta potential of BH suggests increased electrostatic repulsion, which may limit aggregation and help maintain the BH system in a dispersed state. By preserving the accessibility and interfacial area of the active components, this physicochemical property may provide favorable conditions for their interaction with the surrounding biological environment [14]. Nevertheless, colloidal stability should be regarded as a supporting physicochemical property rather than a direct antibacterial or antioxidant mechanism. The biological activity of BH is primarily attributed to the combined contributions of AgNPs-GR, plant-derived phytochemicals, and the chlorophyll-containing biomimetic phospholipid structure. To place the physicochemical characteristics of developed systems in a broader biological context, Table 5 compares the main parameters determined for AgNPs-GR, Lipo and BH with representative green-synthesized, liposomal and lipid-coated Ag-containing nanostructures reported in the literature. The comparison highlights how their physicochemical properties may influence biological and toxicological responses.

The biological and toxicological responses to Ag-containing nanostructures are strongly influenced by several interdependent physicochemical parameters. Kim et al. demonstrated that AgNP toxicity depends on the interplay between primary particle size, surface coating and dispersion state. Although the investigated particles had nominal core sizes of 20 and 110 nm, their hydrodynamic diameters changed markedly with the ionic composition of the exposure medium. When agglomeration was minimized, 20 nm AgNPs were more toxic than 110 nm particles and PVP-coated AgNPs were more toxic than citrate-coated particles, demonstrating that the effective size and dispersion state under biological exposure conditions are critical determinants of AgNP toxicity [49]. El Badawy et al. further demonstrated surface-charge-dependent toxicity of AgNPs toward bacterial cells, emphasizing the importance of nanoparticle surface properties in determining biological interactions [50]. Yang et al. found that both surface coating and dissolved Ag contributed to AgNP toxicity and reported a linear relationship between dissolved Ag and toxicity under their experimental conditions, demonstrating that surface chemistry and dissolution can substantially influence Ag bioavailability and biological response [51].

In green-synthesized systems, Fernandes et al. obtained phytosynthesized AgNPs with an average size of ~89 nm and a zeta potential of −46.2 ± 6.06 mV and showed that the phytogenic system exhibited antimicrobial activity together with a mammalian-cell cytotoxicity profile distinct from chemically synthesized AgNPs [52]. Similarly, Khorrami et al. reported 31.4 nm green AgNPs with a zeta potential of −33.8 mV and demonstrated distinct antimicrobial, antioxidant and cytotoxic effects compared with both the plant extract and commercial AgNPs, supporting the contribution of the phytochemical coating to nanoparticle biological performance [53]. More recent studies reinforce these observations. İpek et al. obtained *Papaver rhoeas*-derived AgNPs of 1.47–7.31 nm with a zeta potential of −36.1 mV and reported both antimicrobial activity and concentration-dependent cytotoxicity toward cancer and healthy HUVEC cells [54]. Jeon et al. reported black-mulberry-derived AgNPs with a hydrodynamic size of 170.17 ± 12.65 nm, PdI of 0.281 ± 0.07 and zeta potential of −56.6 ± 0.56 mV, together with antioxidant and antibacterial effects and an MCF-7 IC_50_ of 96.9 μg/mL [55]. Sahu et al. obtained green-tea-mediated AgNPs with a hydrodynamic diameter of 140.90 nm and zeta potential of −19.7 mV; these particles showed strong antioxidant activity and moderate effects against selected cancer cells while no detectable cytotoxicity was observed in Caco-2 cells under the tested conditions [56]. Likewise, Khan et al. reported *Withania coagulans*-mediated AgNPs with an average size of approximately 37 nm and a zeta potential of −21.4 mV; hemolysis and brine-shrimp assays indicated moderate toxicity, while antioxidant and antibacterial activities were maintained [57].

For the BH system, the association of AgNPs with phospholipid vesicles may further influence nanoparticle–cell interactions and biological response. Barbinta-Patrascu et al. developed multifunctional soft hybrid bio-platforms combining nanosilver with lipid and natural compounds, including chlorophyll *a*, and reported nanoscale architectures with a zeta potential of −30.9 mV together with antioxidant, antibacterial and selective cytotoxic effects [47]. In a subsequent study, Barbinta-Patrascu et al. developed closely related biohybrids based on *Caryophyllus aromaticus*-mediated AgNPs and soybean-lecithin biomimetic membranes. CTEM revealed 6–54 nm clove-generated AgNPs, while incorporation into lipid vesicles increased the magnitude of the zeta potential from −18.9 mV for free AgNPs to −28.0 mV for BH and −31.7 mV for chlorophyll-containing Chla-BH. Importantly, whereas free AgNPs exhibited pronounced cytotoxic and hemolytic effects, both lipid-associated biohybrids showed no significant reduction in L929 fibroblast viability over the tested concentration range and markedly lower hemolysis, demonstrating that association with biomimetic lipid membranes can substantially modify the biological and toxicological response of phytogenic AgNPs [58]. Gorshkova et al. similarly combined phytosynthesized Ag/AgCl nanoparticles with chlorophyll-*a*-labeled soybean-lecithin liposomes. The parent Ag/AgClNPs had a size of 48.2 ± 9.5 nm and a zeta potential of −31.1 ± 1.9 mV, whereas the corresponding biohybrid exhibited a zeta potential of approximately −33 mV and an AFM-derived size of 72.6 ± 18.2 nm. The authors related the observed biological performance to particle size, surface charge, morphology and structural organization [59].

Recent liposomal studies provide further evidence that lipid association can substantially modify AgNP–cell interactions. Skóra et al. developed EGF-labeled AgNP-loaded liposomes with a size of 107.9 nm and a zeta potential of −16.60 mV. Liposomal targeting enhanced the toxic and pro-apoptotic effects toward EGFR-overexpressing cancer cells while reducing toxicity toward normal BJ fibroblasts, illustrating how the lipid carrier and its surface functionality can redirect the biological response of AgNPs [61]. Jayachandran et al. further developed green-synthesized AgNP-loaded liposomes with a hydrodynamic diameter of 172.1 nm, a PdI of 0.381 and a zeta potential of −21.5 mV, illustrating that incorporation of phytogenic AgNPs into a lipid carrier produces physicochemical characteristics in the same general range as the present BH system [62]. Darwish et al. showed that AgNP encapsulation substantially altered the surface charge of phospholipid vesicles; for example, citrate-AgNP-loaded PC/PG liposomes had a hydrodynamic diameter of 104.9 nm and a zeta potential of −37.8 mV, remarkably close to the values obtained for BH in the present study. These Ag–lipid formulations strongly affected melanoma-cell survival, while non-tumor HEK293 cells were less vulnerable to the lipid formulations than to free AgNPs [64].

Other studies further support the importance of lipid association. Yusuf et al. demonstrated that liposomal encapsulation increased AgNP cytotoxicity and altered the mechanism of apoptosis compared with non-encapsulated particles [65], while Yusuf and Casey subsequently showed that liposomal encapsulation enhanced cellular uptake and produced redox imbalance and caspase-dependent apoptosis [66]. Cunningham et al. investigated phosphatidylcholine-containing hybrid lipid-coated AgNPs ranging from 20 to 100 nm and observed size-dependent toxicity even when Ag^+^ release was maintained below 0.2%, demonstrating that particle size itself can remain an important determinant of biological response in lipid-coated systems [60]. Likewise, Lira et al. systematically investigated lipid-coated AgNPs of different sizes and shapes and demonstrated that these structural parameters significantly affected membrane interactions and in vivo toxicity despite comparable surface chemistry [63]. Recently, Rakhy et al. directly examined AgNP interactions with cell-mimetic giant unilamellar vesicles. AgNPs with an average size of 58.1 nm and a zeta potential of −6.9 mV induced concentration-dependent vesicle deformation, nanoscale membrane pore formation, and increased membrane permeability, providing direct evidence that AgNP physicochemical characteristics can influence interactions with lipid membranes [67].

Kessler et al. emphasized that nanoparticle composition, surface chemistry and interactions with physiological media govern ROS-forming reactions relevant to nanotoxicity [68]. These studies support considering particle size, PdI/dispersion state, zeta potential, morphology, phytochemical capping, lipid association and Ag-containing composition as interdependent descriptors when interpreting the biological behavior of AgNPs-GR and BH. However, since direct mammalian cytotoxicity was not evaluated in the present study, these parameters should be regarded as factors potentially relevant to nanotoxicity rather than as direct evidence of an experimentally established toxicity profile.

### 4.2. Investigation of Biological Performance of BH

The antioxidant activity of GR-EV and AgNPs-GR can be associated, at least in part, with the presence of plant-derived bioactive compounds, particularly phenolics and flavonoids [1] whose presence was demonstrated by TPC values (Section 3.4) and by UV–Vis absorption and FT-IR ATR spectra (Section 3.1). During the phytosynthesis process, various biomolecules from the plant extract attach onto the surface of the AgNPs, serving many roles: reducing agents, and also capping and stabilizing the green-synthesized AgNPs. These biomolecules adsorbed on the silver nanoparticle surface (such as polyphenols, flavonoids, proteins, etc.) exhibit intrinsic antioxidant activity, probably due to the presence of biomimetic membranes in the composition of BH, findings that agree with our previous reports [40].

The different antioxidant responses obtained by the CL (Figure 10a) and DPPH (Figure 10b) assays can be related to the distinct reaction conditions and radical species involved in the two methods. The CL assay monitors interactions with short-lived radicals over a short reaction time, whereas the DPPH assay involves a prolonged incubation period that allows equilibration between the radical species and the antioxidants present in the sample. In the present study, AgNPs-GR showed a higher antioxidant response than GR-EV in the CL assay, while a different relative behavior was observed in the DPPH assay. The organization of phytochemicals at the nanoparticle surface may modify their accessibility and reactivity toward different radical species, which could contribute to this assay-dependent behavior. A similar influence of the spatial organization of surface-associated biomolecules on antioxidant response has been proposed previously [69].

On the other hand, antioxidant activity determined by the DPPH method requires an incubation period of approximately 2 h before spectrophotometric measurement, allowing for the equilibration of radical species with the antioxidants present in the sample. This may explain the differing values obtained via the two methods for the plant extract and the AgNPs. In the case of the DPPH method, the spherical surface of AgNPs-GR and the structural rearrangement of the capping agents induce a significant increase in the density of active centers participating in the “equilibration” process, thereby contributing to the modification of the AA% values.

In their review paper, Bedlovičová et al. provided an overview on the antioxidant activity of phyto-derived AgNPs [70]. The authors pointed out that understanding the roles of various antioxidants and their activities is challenging. They highlighted contradictory results from the scientific literature regarding the antioxidant capacity of a plant extract as compared to AgNPs derived from it. Thus, some research groups reported antioxidant capacity values of AgNPs greater than those of vegetal extract itself, while others reported opposite results, pointing out that both scenarios are possible, but a relevant explanation is missing.

In contrast to the variable relative behavior of GR-EV and AgNPs-GR, BH exhibited the highest antioxidant response in both assays. This enhanced activity may result from the combined contributions of the phytochemical-capped AgNPs-GR, chlorophyll, and the biomimetic phospholipid environment. The lipid organization may also modify the local environment and accessibility of the antioxidant components, in agreement with our previous observations for related biohybrid systems [40]. However, since a formal synergy analysis was not performed, the present findings show an enhanced combined antioxidant response.

The GIZD values for AgNPs-GR are similar with those obtained by Riaz et al. who prepared AgNPs using aqueous-root extract of *Jurinea dolomiaea* which showed an inhibition zone diameter of 6.0 mm against *Escherichia coli* [71]. It is worth mentioning that, in our study, the biogenic AgNPs were diluted twice, to achieve the same concentration as in BH.

An increase in antibacterial activity is observed in the case of BH in the agar-diffusion assay, which may be related to the presence of biomimetic membranes that facilitate attachment to bacterial cells, disturbing them. Artificial lipid layers may enhance contact between AgNPs-GR with bacterial membranes and biofilm-associated structures. Panthi et al. [72] pointed out that liposomes have the ability to fuse with biofilm matrices and interact with bacterial cells, resulting in subsequent disruption of the bacterial membrane.

MIC is the lowest concentration of an antimicrobial agent that completely inhibits the visible growth of a microorganism [73]. The lowest MIC values were observed for the *E. coli* Gram-negative strain. For this strain, BH and AgNPs-GR exhibited identical MIC, MBC, and MBEC values, indicating comparable quantitative antibacterial activity under the tested conditions. Against *E. faecalis*, both formulations showed the same MIC and MBEC values, whereas BH exhibited a defined MBC of 250 µL/mL compared with >250 µL/mL for AgNPs-GR. Thus, the enhanced antibacterial response of BH was more evident in the agar-diffusion assay, while the broth-based quantitative response depended on the bacterial strain and evaluated endpoint (Table 4). The greater sensitivity of *E. coli* observed in the present study may be related to structural differences in the bacterial cell wall; while Gram-positive bacteria possess a dense, rigid peptidoglycan layer that can limit the diffusion of nanostructures, Gram-negative bacteria have an outer membrane rich in lipopolysaccharides (LPS) and a thinner peptidoglycan layer [74]. Our results suggest that phytosynthesized nanostructures might interact with the negatively charged LPS-containing outer membrane, facilitating electrostatic interactions and subsequent membrane destabilization and/or intracellular accumulation of the bioactive compounds [75]. Once silver nanoparticles cross the membrane and reach the intracellular space, they can release silver ions (Ag^+^) into the cytoplasm, enhancing the antimicrobial effect by altering the structure or function of vital components [75]. The MBC values (the lowest concentration of a bacterial agent that effectively kills the entire bacterial population) were similar to the MIC values, suggesting a bactericidal mechanism of action for the tested samples. This interpretation has been made according to Morgana et al. [76]; i.e., if the MBC/MIC ratio is ≤4, the effect is considered bactericidal, while if the ratio is >4, the effect is defined as bacteriostatic. Also, the MBEC values (the lowest concentration of compound that inhibits adhesion to an inert substrate) were similar to the MIC and MBC values, suggesting that the biofilm inhibition is the result of bacterial cell killing, and not of the inhibition of biofilm-development-specific mechanisms, such as biofilm matrix production or intercellular communication.

As shown in Table 4, BH and AgNPs-GR exhibited comparable quantitative antibacterial activity against *E. coli*, with identical MIC, MBC and MBEC values. Against *E. faecalis*, BH showed the same MIC as AgNPs-GR but a defined MBC of 250 µL/mL, whereas the MBC of AgNPs-GR remained >250 µL/mL. Thus, the enhancement observed for BH was more evident in the agar-diffusion assay than in the broth-based quantitative assays.

Based on the physicochemical characterizations, AgNPs-GR exhibited a relatively small average particle size (Zav _AgNPs-GR_ = 64.33 ± 9.11 nm), spherical morphology, and a homogeneous and uniform particle size distribution. These characteristics may contribute to the antibiofilm effect observed against the microbial strains evaluated by allowing interaction with the biofilm biomass, increasing the ability to penetrate and cross the biofilm matrix, promoting its disruption and, ultimately, detachment from the inert surface [77]. BH demonstrated superior antibacterial activity, due to its composition and its good physical stability reflected by its most negative zeta potential (see Figure 5). This behavior was highlighted by Belteky et al. [78] who stated that the NPs with the highest colloidal stability, reflected by their most negative zeta, exhibited the strongest antibacterial activity. Due to its high stability, the interaction of BH with the biofilm biomass is achieved by increasing the availability of the active surface area resulting in an enhanced antibiofilm effect [79].

Regarding the modulation of the phenotypic expression of soluble virulence factors, as stated in the methodology section, only the virulence factors for which the untreated control has been positive are commented on, i.e., haemolysin and esculinase for *E. faecalis*, as well as haemolysin, esculinase and caseinase for *E. coli* (Table S3, Supplementary Materials).

In case of the *E. faecalis* Gram-positive strain, the majority of the tested samples decreased the hemolysins production quantified at 24 h, but the inhibitory effect disappeared after 48 h of incubation. It is to be noticed that in the case of GR-EV (1:100), Lipo and Lipo (1:1) the inhibitory effect of this important virulence factor occurred both at MIC/2 and MIC/4, suggesting that at subinhbitory concentrations, these samples could exhibit an anti-pathogenic effect. Most bacterial hemolysins are extracellular proteins classified as invasive factors with a cytolytic role by altering host cell membrane integrity; this mechanism causes erythrocyte lysis and releases hemoglobin and iron, essential elements for pathogen multiplication. They also promote local tissue destruction, induce the inflammatory response, and, in some cases, modulate or suppress the host immune response, thus contributing to the pathogenicity and dissemination of the microorganism [80].

In the case of esculinase, GR-EV exhibited an inhibitory effect at MIC/2, both after 24 and 48 h of incubation. At MIC/4, all tested samples exhibited an inhibitory effect of esculinase production recorded only after 48 h of incubation, an effect also accounting for an antipatogenic effect occurring at subinhibitory concentrations, taking into account that esculinase activity may indicate resilience in hostile environments by promoting colonization and persistence across diverse ecological niches, such as the gastrointestinal [81].

For the Gram-negative *E. coli* strain, Lipo and BH inhibited the amylase production after 48 h of incubation at MIC/4.

These assays have highlighted the anti-virulence potential of the tested samples; however, in certain cases, an overexpression of different soluble enzymes has been recorded in the presence of subinhibitory concentrations of the tested samples that could be explained by the metabolic heterogeneity of the bacterial cultures and the complexity of samples compositions.

The physicochemical properties and bioactivities of the prepared BH are closely correlated. Thus, a more negative zeta potential value correlates with improved antioxidant and antimicrobial activities, due to a better dispersion and enhanced colloidal stability of particles, which facilitates better contact with free radicals and with bacterial cells, respectively. Moreover, the nanoscale size offers more active centers for scavenging free radicals, thus improving antioxidant properties.

In addition, the nanoscale roughness of a material’s surface affects bacterial attachment. So, increased nanoscale roughness induces higher local stress and enhanced bacterial membrane disturbance [82]. In this sense, the antibacterial efficacy increases with increasing of RMS values:

RMS(BH) > RMS(AgNPs-GR) > RMS(Lipo) → GIZD(BH) > GIZD(AgNPs-GR) > GIZD(Lipo)

Overall, BH showed enhanced antioxidant and antibacterial responses compared with the individual building blocks under selected experimental conditions. These effects most likely reflect the combined contribution of AgNPs-GR, plant-derived phytochemicals, chlorophyll, and the biomimetic phospholipid environment. Since no formal synergy analysis was performed, the results are better described as an enhanced combined biological response rather than as evidence of a synergistic effect.

## 5. Conclusions

A botanical hybrid BH was developed by combining *G. robertianum*-mediated AgNPs with chlorophyll-containing soybean lecithin biomimetic membranes. FTIR, EDX, SEM, AFM, DLS, UV–Vis, fluorescence, and zeta-potential analyses showed that BH retained characteristics of both AgNPs-GR and Lipo while exhibiting a distinct structural and physicochemical profile. The simultaneous detection of Ag and phospholipid-associated P, together with the observed spectral, morphological, and colloidal changes, supported the formation of the hybrid system.

BH exhibited the highest antioxidant activity and produced larger inhibition zones against *E. coli* and *E. faecalis* than the composition-matched AgNPs-GR control, whereas GR-EV and Lipo produced no detectable inhibition zones. However, the quantitative antibacterial and antibiofilm assays revealed a more nuanced response, with comparable MIC, MBC, and MBEC values for BH and AgNPs-GR against *E. coli* and only limited differences against *E. faecalis*. Thus, the biological response of BH was dependent on the bacterial strain and experimental endpoint. At subinhibitory concentrations, the tested formulations also modulated the production of selected virulence-associated enzymes, suggesting a potential influence on bacterial pathogenicity-related phenotypes.

Our results indicate that the biological performance of BH arises from the combined contributions of phyto-capped AgNPs-GR, plant-derived compounds, chlorophyll, and the biomimetic phospholipid environment. Since a formal synergy analysis was not performed, the observed effects should be regarded as an enhanced combined response rather than as evidence of synergistic activity. Further studies are needed to clarify the underlying mechanisms, distinguish the respective contributions of the metallic and phytochemical components, and evaluate long-term stability, reproducibility, and mammalian biocompatibility.

## Figures and Tables

**Table 1:** The abbreviations and descriptions of the obtained samples.

Sample Abbreviation	Description
GR-EV	Aqueous vegetal extract of *G. robertianum* L.
AgNPs-GR	Silver nanoparticles phyto-synthesized using GR-EV as a bioreducing agent
Lipo	Chl–Soybean Lecithin–Biomimetic membranes
BH	Botanical hybrid containing Lipo + AgNPs-GR (1:1, *v*/*v*)

**Table 2:** Roughness (RMS) values of the bio-based architectures prepared in this work.

Sample	RMS (nm)
AgNPs-GR	9.52
Lipo	1.939
BH	10.69

**Table 3:** The growth inhibition zone diameters (GIZDs) obtained for BH/constituent components against the two reference bacterial strains (*E. coli* ATCC 25922 and *E. faecalis* ATCC 29212). The building blocks of BH are in the same amounts as in BH.

Samples	Strains	GIZD (mm)
GR-EV	*E. faecalis*	0.00 ± 0.00
AgNPs-GR	*E. faecalis*	6.50 ± 0.71
Lipo	*E. faecalis*	0.00 ± 0.00
BH	*E. faecalis*	10.00 ± 0.00
GR-EV	*E. coli*	0.00 ± 0.00
AgNPs-GR	*E. coli*	5.50 ± 0.71
Lipo	*E. coli*	0.00 ± 0.00
BH	*E. coli*	10.00 ± 0.00

**Table 4:** MIC, MBC and MBEC values obtained for BH components against the two tested bacterial strains (*E. coli* ATCC 25922 and *E. faecalis* ATCC 29212). The building blocks of BH are in the same amounts as in BH.

Samples	Strains	MIC (µL/mL)	MBC (µL/mL)	MBEC (µL/mL)
GR-EV	*E. faecalis*	>500	>500	>500
AgNPs-GR	*E. faecalis*	250	>250	250
Lipo	*E. faecalis*	>500	>500	>500
BH	*E. faecalis*	250	250	250
GR-EV	*E. coli*	>500	>500	>500
AgNPs-GR	*E. coli*	125	125	125
Lipo	*E. coli*	>500	>500	>500
BH	*E. coli*	125	125	125

Note The quantitative assays have been performed using a range of serial two-fold concentrations, from 500 to 1 mg/mL. When no growth inhibition has been observed, the MIC could not be established due to being higher than the highest tested concentration; these cases are presented in the table as >500.

**Table 5:** Physicochemical characteristics of the developed nanosystems compared with representative Ag-containing systems reported in the literature and their biological/toxicological responses.

Surface/Formulation	Size (nm)	PdI	Zeta Potential (mV)	Reported Biological/Toxicological Response	Reference
AgNPs-GR—present study Quasi-spherical phytosynthesized AgNPs; *G. robertianum*-derived phytochemical capping; RMS 9.52 nm	64.33 ± 9.11, DLS	≈0.15	−24.8	Antioxidant activity: 61.19% CL; 37.23% DPPH; GIZD 6.50 ± 0.71 mm (*E. faecalis*) and 5.50 ± 0.71 mm (*E. coli*); mammalian cytotoxicity not evaluated	
Lipo—present study Soy-lecithin/chlorophyll phospholipid vesicles; RMS 1.939 nm	139.70 ± 13.12, DLS	≈0.28	−16.5	Low antioxidant activity and no detectable inhibition zone against either bacterial strain under the tested conditions; mammalian cytotoxicity not evaluated	
BH—present study AgNPs-GR associated with soy-lecithin/chlorophyll biomimetic membranes; RMS 10.69 nm	125.40 ± 3.15, DLS	≈0.31	−37.5	Highest antioxidant response: 93.84% CL; 79.22% DPPH; GIZD 10.00 ± 0.00 mm against both strains; mammalian cytotoxicity not evaluated	
PVP- or citrate-coated AgNPs; dispersion controlled by ionic strength	20 and 110, core size	NR	Negative for all formulations; numerical values not reported	When agglomeration was controlled, 20 nm AgNPs were more toxic than 110 nm AgNPs and PVP-coated particles were more toxic than citrate-coated particles. Increased hydrodynamic diameter/agglomeration in embryo medium reduced bioavailability and toxicity.	[49]
Uncoated, citrate-, PVP- and BPEI-coated AgNPs	HDD (nm): 18, 10, 12, 10 for uncoated (H_2_-) or coated (with citrate-, PVP-, and BPEI-) AgNPs	NR	−22, −38, −10, +40, respectively	Strong surface-charge dependence of antibacterial toxicity; positively charged BPEI-AgNPs were the most toxic, while strongly negative citrate-AgNPs were the least toxic.	[50]
Citrate-, PVP- and gum-arabic-coated AgNPs	5, 8, 17, 22, 38, plus other sizes	NR	Coating-dependent	*C. elegans* EC_50_ values ranged approximately 0.6–463 μM depending on coating and medium. Toxicity correlated with dissolved Ag and coating, but not simply with particle size.	[51]
*Punica granatum* peel-extract-mediated AgNPs; phytocompounds acting as reducing/stabilizing agents	89 ± 21, DLS *(green formulation)*; conventional formulation: 19 ± 4, DLS	NR	−46.2 ± 6.06 (green AgNPs); conventional AgNPs: −67.5 ± 3.69	Green AgNPs showed markedly lower cytotoxicity toward L929 fibroblasts than conventionally synthesized AgNPs. Toxicity of green AgNPs became evident at 50 μg/mL, with cell viability still close to 80%, whereas conventional AgNPs produced <20% viability at 6.25 μg/mL. The authors suggested that phytochemical stabilization and reduced Ag^+^ release may contribute to the lower toxicity of the green system.	[52]
Walnut-green-husk-derived biological coating	31.4	NR	−33.8	At 60 μg/mL, green AgNPs caused ~70% cytotoxicity in MCF-7 cells versus ~56% for commercial AgNPs and ~40% for plant extract; lower toxicity was observed toward L929 cells.	[53]
*Papaver rhoeas* leaf-extract-mediated, mainly spherical AgNPs	1.47–7.31	NR	−36.1	Strong antimicrobial activity; IC_50_ approximately 2.37–3.53 μg/mL depending on cell line and exposure time, including both cancer cells and healthy HUVEC cells.	[54]
*Black-mulberry*-extract-mediated AgNPs	170.17 ± 12.65	0.281 ± 0.07	−56.6 ± 0.56	Improved antioxidant activity; MIC 600 μg/mL against *S. aureus* and *E. coli*; MCF-7 IC_50_ 96.9 μg/mL.	[55]
Green-tea-extract-mediated AgNPs	140.90, DLS; 9 ± 0.025, TEM; 20.27 ± 1, SEM	25.8% (≈0.258)	−19.7	Strong antioxidant activity; moderate effects against KB3 and AGS cancer cells, with no detectable cytotoxicity in Caco-2 cells under the reported conditions.	[56]
*Withania coagulans* leaf-extract-mediated AgNPs	≈37, reported average; 26.63, SEM/HRTEM	NR	−21.4	Antioxidant and antibacterial activity; hemolysis IC_50_ 141.466 μg/mL and brine-shrimp lethality IC_50_ 721.76 μg/mL, interpreted as moderate toxicity.	[57]
Nano-Ag + lipids + chitosan + chlorophyll *a* + curcumin	<200, DLS/AFM	NR	−30.9	Antioxidant activity 96.63%; *E. coli* inhibition zone 32 mm; cytotoxic toward HT-29 cells but no toxicity to normal cells over the reported 5.7–17% concentration range; hemocompatible.	[47]
*Caryophyllus aromaticus*-mediated AgNPs capped by clove-derived proteins/polysaccharides/phytochemicals; associated with soybean-lecithin biomimetic membranes ± chlorophyll a	CE-nAg: 6–54, CTEM; BH and Chla-BH: nanoscale, with broader distribution/agglomeration for BH and narrower distribution for Chla-BH	NR	CE-nAg −18.9; Lip −12.1; Chla-Lip −16.1; BH −28.0; Chla-BH −31.7	CE-nAg: 90.24% antioxidant activity, *E. coli* ZOI 18 mm, HT-29 IC_50_ 3.6% *v*/*v*, and 89% hemolysis at 33.4% *v*/*v*. BH/Chla-BH showed higher antioxidant activity (92.62/98.8%) and ZOI of 25/42 mm, respectively. Both BHs showed no significant reduction in L929 viability over 0–22.79% *v*/*v* and very low hemolysis (0.46 ± 0.30% BH; 1.68 ± 0.14% Chla-BH), indicating markedly improved hemocompatibility compared with free CE-nAg.	[58]
Green Ag/AgClNPs associated with chlorophyll-*a*-labeled soybean-lecithin liposomes	Ag/AgClNPs 48.2 ± 9.5; BH P5 72.6 ± 18.2 AFM/76.3 SAXS	NR	Ag/AgClNPs −31.1 ± 1.9	Antioxidant, antimicrobial and antiproliferative activity; non-hemolytic below 102.2 μM.	[59]
PC/sodium-oleate/hexanethiol hybrid lipid coating	20, 40, 60, 80, 100	NR	NR	Ag^+^ release <0.2%. Significant zebrafish mortality started at 3 mg/L for 20/40 nm and 4 mg/L for 60 nm particles; 80 and 100 nm particles produced no significant mortality, demonstrating size-dependent toxicity.	[60]
AgNPs entrapped in EGF-labeled liposomes	107.9	NR	−16.60	Greater toxicity and pro-apoptotic effects in EGFR-overexpressing A549/SCC-15 cancer cells than free AgNPs, but lower toxicity toward normal BJ fibroblasts; ROS-dependent effects were observed.	[61]
Green-synthesized AgNPs encapsulated in liposomes	172.1, DLS; 80–97, FESEM	0.381	−21.5	Encapsulation efficiency 82.25%; release 73.32 ± 0.68% at 72 h at pH 5.5; useful close physicochemical comparator, although direct mammalian cytotoxicity was not assessed.	[62]
Constant HPC/sodium-oleate/hexanethiol hybrid lipid surface	Spheres 5–100; plates 40–100; cubes 55–100	NR	NR	Size and shape significantly influenced membrane interactions and zebrafish toxicity. Significant mortality occurred particularly for 5, 20, 40 and 60 nm spherical particles at ≥6 mg Ag/L.	[63]
Citrate-capped AgNPs associated with PC/PG phospholipid vesicles	104.9, DLS	NR	−37.8	Ag–lipid formulations strongly reduced melanoma-cell viability; non-tumor HEK293 cells were less affected by liposomal formulations than by free AgNPs. The size/ζ values are notably close to BH.	[64]
AgNPs encapsulated in DPPC liposomes	NR	NR	NR	Lipo-AgNPs induced significant THP-1 cell death at 0.625 μg/mL, lower than the dose required for uncoated AgNPs, and altered the apoptotic mechanism.	[65]
DPPC-liposomal AgNPs	NR	NR	NR	Liposomal encapsulation increased cellular uptake, cytotoxicity and DNA damage and produced redox imbalance with caspase-dependent apoptosis relative to free AgNPs.	[66]
AgNPs interacting with cell-mimetic giant unilamellar lipid vesicles	58.1	NR	−6.9	At 0.3–5.0 μg/mL, AgNPs caused concentration-dependent vesicle deformation, nanoscale pore formation and increased membrane permeability, directly demonstrating physicochemical AgNP–lipid membrane interactions.	[67]

Note: NR, not reported or not available as a directly comparable value. Literature values are provided for physicochemical and mechanistic context rather than direct quantitative comparison, since synthesis routes, media, concentrations, biological models and toxicity endpoints differ among studies. HDD—Hydrodynamic diameter, PVP—polyvinylpyrrolidone, BPEI—branched polyethyleneimine, GUV—giant unilamellar vesicle, DPPC—Dipalmitoylphosphatidylcholine, PC—Phosphatidylcholine, PG—Phosphatidylglycerol, CE—clove extract, EGF—epidermal growth factor, EGFR—epidermal growth factor receptor, HPC—hydrogenated phosphatidylcholine, ROS—reactive oxygen species.

## Data Availability

The original contributions presented in this study are included in the article/Supplementary Materials. Further inquiries can be directed to the corresponding author.

## Supplementary Materials

The following supporting information can be downloaded at: https://www.mdpi.com/article/10.3390/biomimetics11100713/s1, Table S1: FT-IR ATR band assignment for the prepared samples; Table S2: Mean elemental composition determined by EDX analysis (at.%, mean ± SD, n = 3, ND = not detected); Table S3: Screening of the anti-virulence potential of the tested samples by modulating exoenzymatic profiles in *Enterococcus faecalis* and *Escherichia coli* at subinhibitory concentrations (µL/mL). The colors used indicate the following: green—complete and reduced inhibition of enzymatic activity; yellow—stimulation of enzymatic activity; orange—activity similar to the strain control; red—stimulation of enzymatic activity.
