# Supplementary material for: Botanical Hybrid BioEntities with Antibacterial Activity Derived from Geranium robertianum—Biophysical Insights
Source: Biomimetics (Basel). 2026 Oct 6;11(10):713. doi: 10.3390/biomimetics11100713 (PMC13702121; doi:10.3390/biomimetics11100713)
Supplement: Supplementary file 1 [file biomimetics-11-00713-s001.zip › biomimetics-4525370-supplementary.pdf]

Supplementary Material

Article

Botanical Hybrid BioEntities with Antibacterial Activity Derived from *Geranium robertianum*—Biophysical Insights

Marcela-Elisabeta Barbinta-Patrascu [1], Irina Negut [2,*], Cornelia Nichita [3,4], Bogdan Bita [1,5], Vlad-Andrei Antohe [1], Georgiana-Alexandra Grigore [6,7,8], Diana-Madalina Gaboreanu [6,7,8] and Adrian Bobica [9]

1 Department of Electricity, Solid-State Physics and Biophysics, Faculty of Physics, University of Bucharest, 405 Atomistilor, 077125 Magurele, Romania; marcela.barbinta@unibuc.ro (M.-E.B.-P.); bogdan.bita@inoe.ro (B.B.); vlad.antohe@fizica.unibuc.ro (V.-A.A.)
2 National Institute for Laser, Plasma and Radiation Physics, 409 Atomistilor, 077125 Magurele, Romania
3 CTT-3Nano-SAE Research Center, Faculty of Physics, University of Bucharest, ICUB, 405 Atomistilor Street, P.O. Box MG-38, 077125 Magurele, Romania; cornelia.nichita@unibuc.ro
4 National Institute for Chemical—Pharmaceutical Research and Development, 112 Vitan Avenue, 031299 Bucharest, Romania
5 Optospintronics Department, National Institute of Research and Development for Optoelectronics-INOE 2000, 409 Atomistilor, 077125 Magurele, Romania
6 Faculty of Biology, University of Bucharest, Splaiul Independentei 91–95, 050095 Bucharest, Romania; grigore.georgiana-alexandra@s.bio.unibuc.ro (G.-A.G.); gaboreanu.diana-madalina@s.bio.unibuc.ro (D.-M.G.)
7 Research Institute of the University of Bucharest—ICUB, University of Bucharest, 050663 Bucharest, Romania
8 National Institute of Research and Development for Biological Sciences, Splaiul Independentei 296, 060031 Bucharest, Romania
9 CROMATEC PLUS SRL, 1 Petre Ispirescu Street, Tâncăbeşti, 077167 Ilfov, Romania; adrian.bobica@cromatecplus.ro
* Correspondence: negut.irina@inflpr.ro

Table S1. FT-IR ATR band assignment for the prepared samples

Sample	FT-IR Bands (cm^{-1})	Assignment	Ref.
Lipo	3189 (broad, strong)	O–H stretching in water molecules tightly associated with the biomimetic membranes *via* hydrogen bonds	[1,2]
	2935 (weak)	C–H anti-symmetric stretching vibration in the CH_2 groups of alkyl chains of lipids	[2]
	2851 (weak)	C–H symmetrical stretch vibration of alkyl chains	[2]
	1645 (narrow, medium)	Stretching vibrations of the ester carbonyl groups of lipids	[1]
	1297 (broad, weak)	Antisymmetric PO_2^- stretching in lipids	[1]
	1078 (sharp, strong)	Symmetric PO_2^- stretching in lipids; Antisymmetric stretching of –C–O group of chlorophyll	[1,3]
	973 (medium)	Antisymmetric choline group ($N^+ - (CH_3)_3$) stretching vibrations in lipids	[1]
	853 (weak)	Stretching vibrations of $-CH_2-$ in lipids	[1]
GR-EV	3270 (sharp,	O–H bending and stretching vibrations of hydroxyl groups belonging to phenolic compounds	[2,3]

	narrow, strong)	and polysaccharides; Stretching vibrations of the primary and secondary amines	
	2935 (weak)	C–H anti-symmetric stretching vibration	[2]
	2851 (shoulder)	C–H symmetrical stretch vibration of alkyl chains	[2]
	1707 (sharp, weak)	Amide I, due to carbonyl stretch in proteins; Carboxylate groups (–COO^-); Stretching C=C (aromatic ring); Secondary amine, N–H bend	[2,3]
	1594 (strong, sharp)	Amide II due to N–H bending and C–N stretching in proteins	[3-5]
	1391 (strong, sharp)	Carboxylates; Phenol or tertiary alcohol, O–H bend; Primary or secondary, O–H in-plane bend; C–H bend	[2]
	1231 (weak)	Primary or secondary O–H in-plan bend; Aromatic ethers, aryl–O stretch; Aromatic primary amine, C–N stretch	[2]
	1064 (strong, sharp)	Cyclic ethers, large rings, C–O stretch; –C–O–C– ether<br>Antisymmetric stretching of –C–O group of polysaccharides and/or chlorophyll;<br>C–O bending in esters; Secondary alcohol, C–O stretch; Primary amine, C–N stretch	[2,3]
**AgNPs-GR**	3265 (broad)	O–H stretching alcohols, polysaccharides and phenolic compounds/ polyphenols; Stretching vibrations of the primary and secondary amines	[2,3]
	2935 (weak)	C–H anti-symmetric stretching vibration	[2]
	2851 (weak)	C–H symmetrical stretch vibration of alkyl chains	[2]
	1718 (weak)	Amide I, due to carbonyl stretch in proteins; Carboxylate groups (–COO^-);<br>Stretching C=C (aromatic ring); Primary amine, N–H bend	[2,3]
	1617 (weak, broad)	Amide II, due to N–H bending and C–N stretching in proteins	[3-5]
	1347 (medium,broad)	Carboxylates; Phenol or tertiary alcohol, O–H bend; Primary or secondary, O–H in-plane bend; C–H bend; Amide III band in proteins	[2,3]
	1057 (weak, broad)	Cyclic ethers, large rings, C–O stretch; –C–O–C– ether<br>Antisymmetric stretching of –C–O group of polysaccharides and/or chlorophyll;<br>C–O bending in esters; Secondary alcohol, C–O stretch; Primary amine, C–N stretch	[2,3]
**BH**	3164 (very broad)	Very broad band overlapping the frequencies of the following groups: Hydrogen-bonded O–H (the bending and stretching vibrations of hydroxyl groups in alcohols, polysaccharides and phenolic compounds)	[2,3]
	2935 (weak, sharp)	C–H anti-symmetric stretching vibration; Transition metal carbonyls	[2]
	2851 (weak, sharp)	C–H symmetrical stretch vibration of alkyl chains	[2]
	1653 (broad, weak)	Amide I, arising due to carbonyl stretch in proteins; Carboxylate groups (–COO^-);<br>Stretching C=C (aromatic ring); Primary amine, N–H bend	[2,3]
	1391 (medium, broad)	Carboxylates; Phenol or tertiary alcohol, O–H bend; Primary or secondary, O–H in-plan bend; C–H bend; Amide III band in proteins	[2,3]
	1082 (medium, narrow)	–C–O–C stretching vibration; Primary alcohol, C–O stretch; Antisymmetric stretching of –C–O group of chlorophyll	[2,6]

	980 (weak)	Antisymmetric choline group (N+ – (CH3)3) stretching vibrations in lipids	[1]
	861 (weak)	Hydrogen-bonded O–H out-of-plane bending	[2]

Table S2. Mean elemental composition determined by EDX analysis (at.%, mean ± SD, n = 3, ND = not detected).

Element	GR-EV	AgNPs-GR	Lipo	BH
C	40.03 ± 0.47	46.60 ± 3.24	16.24 ± 8.03	35.89 ± 3.26
N	3.66 ± 0.47	3.23 ± 1.71	0.43 ± 0.20	2.85 ± 1.22
O	44.67 ± 0.47	10.29 ± 2.59	30.75 ± 2.62	23.18 ± 6.80
Na	1.11 ± 0.01	0.46 ± 0.07	18.86 ± 3.43	12.94 ± 3.78
Mg	0.81 ± 0.04	0.26 ± 0.02	0.44 ± 0.06	0.31 ± 0.22
P	0.71 ± 0.12	0.41 ± 0.09	7.45 ± 2.46	5.29 ± 2.55
Cl	2.42 ± 1.27	0.12 ± 0.02	3.65 ± 1.93	1.98 ± 0.71
Ag	ND	0.30 ± 0.33	ND	0.13 ± 0.059
K	4.73 ± 1.10	0.14 ± 0.04	1.11 ± 0.32	0.16 ± 0.10

Note: The Si signal attributed to the underlying substrate was omitted from Table S2 and excluded from the interpretation of the sample composition. The values reported in Table S2 are mean atomic percentages.

Table S3. Screening of the anti-virulence potential of the tested samples by modulating exoenzymatic profiles in *Enterococcus faecalis* and *Escherichia coli* at subinhibitory concentrations (µL/mL). The colors used indicate: green – complete and reduced inhibition of enzymatic activity; yellow – stimulation of enzymatic activity; orange – activity similar to the strain control; red – stimulation of enzymatic activity. The building blocks of BH are in the same amounts as in the biohybrid.

**Compound/Reference tested strain 24h**	**Hemolysin**						**Esculinase**					
	**MIC/2 (µL/mL)**			**MIC/4 (µL/mL)**			**MIC/2 (µL/mL)**			**MIC/4 (µL/mL)**		
	**Mean**	**Std. dev.**	**p-value**	**Mean**	**Std. dev.**	**p-value**	**Mean**	**Std. dev.**	**p-value**	**Mean**	**Std. dev.**	**p-value**
E.faecalis	**100**	**47.14**	**-**	**100**	**0**	**-**	100	23.57	-	100	0	-
GR-EV	0	0	0	0	0	0	83.33	0	0.99	137.5	17.68	0.59
AgNPs-GR	66.67	0	0.94	100	0	0.05	91.67	11.78	>0.99	162.5	17.68	0.08
Lipo	0	0	0	0	0	0	108.33	11.78	>0.9999	112.5	53.03	0.99
BH	66.67	0	0.94	100	0	0.05	125	11.78	0.91	150	0	0.24

**Compound/Ref**	**Hemolysin**	**Esculinase**

erence tested strain 48h	MIC/2 (µL/mL)			MIC/4 (µL/mL)			MIC/2 (µL/mL)			MIC/4 (µL/mL)		
	Mean	Std. dev.	p-value	Mean	Std. dev.	p-value	Mean	Std. dev.	p-value	Mean	Std. dev.	p-value
E.faecalis	**100**	**0**	**-**	**100**	**0**	**-**	100	0	-	100	0	-
GR-EV	100	0	>0.99	100	0	0.05	150	30.30458	0.0012	60.71429	5.0507	<0.0001
AgNPs-GR	100	0	>0.99	100	0	0.05	150	10.10153	0.0012	64.28571	10.1015	<0.0001
Lipo	100	0	>0.99	100	0	0.05	150	10.10153	0.0012	57.14286	0	<0.0001
BH	100	0	>0.99	100	0	0.05	150	10.10153	0.0012	71.42857	10.1015	0.0001

Compound/Reference tested strain 24h	Hemolysin						Caseinase					
	MIC/2 (µL/mL)			MIC/4 (µL/mL)			MIC/2 (µL/mL)			MIC/4 (µL/mL)		
	Mean	Std. dev.	p-value	Mean	Std. dev.	p-value	Mean	Std. dev.	p-value	Mean	Std. dev.	p-value
E.coli	**100**	0	**-**	**100**	**0**	**-**	**100**	0	-	**100**	0	-
GR-EV	100	0	0.05	100	0	0.05	150	70.71	0.86	100	0	0.05
AgNPs-GR	100	0	0.05	100	0	0.05	150	70.71	0.86	100	0	0.05
Lipo	100	0	0.05	100	0	0.05	150	70.71	0.86	100	0	0.05
BH	100	0	0.05	100	0	0.05	100	0	>0.99	100	0	0.05

Compound/Reference tested strain 48h	Hemolysin						Esculinase			Amylase		
	MIC/2 (µL/mL)			MIC/4 (µL/mL)			MIC/4 (µL/mL)			MIC/4 (µL/mL)		
	Mean	Std. dev.	p-value	Mean	Std. dev.	p-value	Mean	Std. dev.	p-value	Mean	Std. dev.	p-value
E.coli	**100**	0	**-**	**100**	**0**	**-**	**100**	28.28427	-	**100**	47.14045	-
GR-EV	100	0	0.05	100	0	0.05	0	0	0	0	0	0
AgNPs-GR	100	0	0.05	100	0	0.05	0	0	0	0	0	0
Lipo	100	0	0.05	100	0	0.05	0	0	0	133.3333	0	0.9104
BH	100	0	0.05	100	0	0.05	160	0	0.7073	66.6667	0	0.9104

References

1. Pawlikowska-Pawlega, B.; Kapral, J.; Gawron, A.; Stochmal, A.; Zuchowski, J.; Pecio, L.; Luchowski, R.; Grudzinski, W.; Gruszecki, W.I. Interaction of a quercetin derivative - lensoside Aβ with liposomal membranes, *Biochim. Biophys. Acta Biomembr*. **2018**, *1860*, 292–299. https://doi.org/10.1016/j.bbamem.2017.10.027
2. Miller, L.M.; Coates, J.P. Interpretation of Infrared Spectra: A Practical and Systematic Approach. In *Encyclopedia of Analytical Chemistry*; John Wiley & Sons, Ltd, 2025; pp. 1–24 ISBN 978-0-470-02731-8.
3. Barbinta-Patrascu, M.-E.; Chilom, C.; Nichita, C.; Zgura, I.; Iftimie, S.; Antohe, S. Biophysical insights on Jack bean urease in the presence of silver chloride phytonanoparticles generated from *Mentha piperita* L. leaves, *Rom.Rep.Phys*. **2022**, *74*, 605.
4. Kiseleva, A.; Nestor, G.; Östman, J. R.; Kriuchkova, A.; Savin, A.; Krivoshapkin, P.; Krivoshapkina, E.; Seisenbaeva, G. A.; Kessler, V. G. Modulating Surface Properties of the *Linothele fallax* Spider Web by Solvent Treatment, *Biomacromolecules* **2021**, *22*, 4945-4955. https://doi.org/10.1021/acs.biomac.1c00787
5. Goto, H.; Kikuchi, R.; Wang, A. Spider Silk/Polyaniline Composite Wire, *Fibers* **2016**, *4(2)*, 12. https://doi.org/10.3390/fib4020012

6. Gorinstein, S.; Park, Y.S.; Heo, B.G.; Namiesnik, J.; Leontowicz, H.; Leontowicz, M.; Ham, K.S.; Cho, J.Y.; Kang, S.G. A comparative study of phenolic compounds and antioxidant and antiproliferative activities in frequently consumed raw vegetables, *Eur. Food Res. Technol*. **2009**, *228*, 903-911. https://doi.org/10.1007/s00217- 008-1003-y.

Disclaimer/Publisher's Note: The statements, opinions and data contained in all publications are solely those of the individual author(s) and contributor(s) and not of MDPI and/or the editor(s). MDPI and/or the editor(s) disclaim responsibility for any injury to people or property resulting from any ideas, methods, instructions or products referred to in the content.
